# DES-Based Green Synthesis of 2D Fluorescent Molybdenum Disulfide for Highly Sensitive and Selective Detection of Nifedipine

**DOI:** 10.1021/acs.analchem.6c02269

**Published:** 2026-10-01

**Authors:** Rafee Ullah, Shams Ur Rehman, Adnan Khan, Hui-Fen Wu

**Affiliations:** † Department of Chemistry, National Sun Yat-Sen University, Kaohsiung, 70, Lien-Hai Road, Kaohsiung 80424, Taiwan; ‡ School of Pharmacy, College of Pharmacy, Kaohsiung Medical University, Kaohsiung 807, Taiwan; § School of Medicine, College of Medicine, National Sun Yat-Sen University, Kaohsiung 80424, Taiwan; ∥ Institute of Medical Science and Technology, National Sun Yat-Sen University, Kaohsiung 80424, Taiwan; ⊥ Institute of Precision Medicine, National Sun Yat-Sen University, Kaohsiung 80424, Taiwan; # Institute of Bio Pharmaceutical Science, National Sun Yat-Sen University, Kaohsiung 80424, Taiwan

## Abstract

Highly fluorescent 2D MoS_2–x_ nanosheets exhibiting intense blue emission were synthesized using a green chemistry approach, utilizing DES as both the reaction medium and a surface passivating agent. The material was synthesized via a hydrothermal method, followed by ultrasonication-assisted exfoliation to yield ultrathin nanosheets with enhanced photoluminescence properties. The 2D MoS_2–x_ nanosheets demonstrated strong fluorescence at λ_em_ = 420 nm when excited at λ_ex_ = 330 nm. These nanosheets were employed as a fluorescence probe for the detection of the calcium channel blocker drug nifedipine (NIF) via a fluorescence quenching mechanism. Upon addition of NIF, the fluorescence of the 2D MoS_2–x_ nanosheets was quenched due to electron transfer from the nanosheets to the electron-deficient NIF molecules, combined with the inner filter effect (IFE). The nanosheets enabled highly sensitive and selective detection of NIF, with photoluminescence spectroscopy revealing a broad linear detection range of 0.6–360 nM (R^2^ = 0.993) and an exceptionally low limit of detection (LOD) of 0.40 nM. The system showed high selectivity toward NIF over structurally similar compounds, drugs, and common metal ions. The sensor was effectively applied for the detection of NIF in urine and Lotus Pond water samples, highlighting its potential for practical pharmacological monitoring. This study presents a sustainable and effective approach for fabricating DES-assisted 2D nanosheets for trace-level analyte detection.

## Graphical abstract

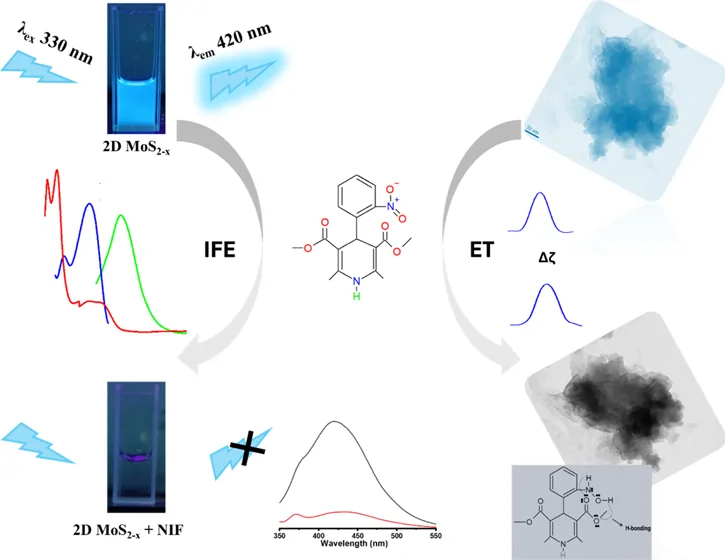

## Introduction

Cardiovascular diseases are the primary cause of mortality globally, with hypertension identified as one of the most critical contributing risk factors.^1,2^ Hypertension is a chronic cardiovascular disorder diagnosed when arterial blood pressure consistently reaches or exceeds 140 mmHg systolic or 90 mmHg diastolic.^3^ Individuals with hypertension are commonly treated with pharmacological agents such as calcium channel blockers, angiotensin-converting enzyme (ACE) inhibitors, vasodilators, and thiazide diuretics to achieve blood pressure control.^1,4^ Nifedipine (dimethyl 1,4-dihydro-2,6-dimethyl-4-(2-nitrophenyl) pyridine-3,5-dicarboxylate) is a dihydropyridine-class calcium channel blocker that is extensively used in the treatment of essential hypertension, congestive heart failure, and ischemic disorders.^5^ Although nifedipine (NIF) is primarily utilized to treat high blood pressure and angina, it also has off-label clinical applications in managing Raynaud’s phenomenon, preventing premature labor, and alleviating painful esophageal spasms.^6^ NIF acts by blocking the flow of L-type calcium ions into smooth muscles of blood vessels. This relaxes coronary and systemic arteries and prevents calcium from causing vasoconstriction. Although NIF is therapeutically valuable, overdose may induce hypotension and reflex tachycardia, along with side effects such as dizziness, palpitations, constipation, and tachycardia.^7^ NIF enters aquatic environments through pharmaceutical waste, improper disposal of unused medications, and human waste. It can persist and accumulate, posing harm to aquatic organisms by disrupting their normal functions even at low concentrations. Therefore, diligent monitoring of nifedipine levels in water is essential.^7,8^

Detection of NIF is critical since it often co-occurs in biological and environmental matrices. Several techniques have been employed for NIF detection, including UV–vis spectroscopy, mass spectrometry, high-performance liquid chromatography (HPLC),^9,10^ surface-enhanced Raman spectroscopy (SERS),^9^ and capillary electrophoresis.^10^ However, these techniques have certain limitations, including inadequate robustness, costly apparatus, limited signal-to-noise ratios, high expenses, intricate sample preparation, protracted analysis duration, and unsuitability for real-time or on-site analysis.^8,11^ Optical and electrochemical sensors have emerged as quick, cost-effective alternatives to conventional methods. However, electrochemical sensors often suffer from limited selectivity in real-sample analyses.^8^ Interfering biomolecules with oxidation potentials similar to nifedipine can influence the selectivity of electrochemical sensors. In contrast, fluorescence-based sensors offer superior sensitivity, selectivity, affordability, rapid response, and operational simplicity, making them more suitable for analyses in complex matrices.^12^ Fluorescence-based recognition has evolved as a popular technique due to its quick response, superior sensitivity, low cost, and suitability for real-time, on-site analysis.^13,14^ Fluorescent sensors commonly function through mechanisms such as fluorescence resonance energy transfer (FRET), photoinduced electron transfer (PET), and intramolecular charge transfer (ICT) to detect analytes selectively and in real time.^15^ These probes are specifically useful in environmental and biological applications, as they allow the detection of trace analytes without requiring considerable sample pretreatment.^16^

Nanomaterials, defined as materials with at least one dimension in the 1–100 nm range, exhibit properties different from their bulk counterparts. They are often classified as zero-dimensional (0D), one-dimensional (1D), two-dimensional (2D), or three-dimensional (3D) according to their structural dimensions.^17^ 2D materials made from abundant elements such as graphene, phosphorene, and carbon nitrides are attractive alternatives to metal-based materials for applications in light-based technologies, sensing, and catalysis.^18^ However, challenges such as complex synthesis and limited stability hinder their application.^19^ Emerging materials like silicene and tellurene offer alternatives, though issues of scalability and durability persist. Elemental sulfur, owing to its natural abundance, low cost, and minimal environmental impact, represents a promising candidate for sustainable materials development.^20^ Recent studies by Mandal et al. have highlighted how structural modifications of sulfur dots significantly influence their photophysical and photochemical behaviors.^21^ Compared to 0D sulfur dots, well-defined 2D sulfur-based layered structures offer advantages such as enhanced surface area, tunable band gaps, lower exciton binding energies, and improved charge separation.^18,22^

The increasing demand for environmentally friendly nanomaterial synthesis has made deep eutectic solvents (DESs) promising green and useful solvents that not only serve as solvents for material synthesis but also help in tuning the fluorescence properties of the resulting nanosheets. These hydrogen bond donor–acceptor-based solvents possess low toxicity, are biodegradable, and offer tunable properties, supporting their application in green nanomaterial fabrication.^23^ The physicochemical properties of DESs (viscosity, polarity, surface tension) and hydrogen bonding capability, together with interactions with the precursors and surfaces, critically influence species reactivity and mass transport, thereby controlling nanostructure formation. Moreover, DES components actively participate in nucleation and growth by neutralizing charges, altering reduction potentials, and passivating certain crystal facets. This ultimately directs growth in preferred crystallographic directions.^24^ Beyond their role in synthesis, DES-derived nanomaterials exhibit enhanced functionality, particularly in sensing applications. DES-based systems enable the fabrication of functionalized nanostructures for detecting heavy metals, gases, biomolecules, and food contaminants. Their hydrogen-bonding environment stabilizes intermediates and promotes the formation of active sites, improving sensitivity and selectivity. Furthermore, synergistic interactions between DESs and nanomaterials, such as metal nanoparticles, enhance analytical performance, enabling low detection limits and efficient extraction in complex matrices.^25^ An additional benefit is that they are significantly cheaper than ionic liquids (ILs).^26^ The adjustable composition of hydrogen bond donors and acceptors in DESs as an active solvent allows fine-tuning of their physicochemical properties, providing a distinct advantage for their use as tailored solvents in nanomaterial synthesis.^27^ The integration of DESs into nanomaterial synthesis draws on established principles from conventional aqueous-based methods, including electrodeposition, solvothermal synthesis, calcination, and Pickering emulsion polymerization.^28^ This approach can be applied to DES systems, providing clear benefits compared to traditional methods.^29^ Ongoing advancements in eutectic systems continue to broaden the scope for innovative approaches in nanomaterial synthesis. Over the last ten years, there has been growing interest in using DESs for the fabrication of nanomaterials for a broad spectrum of applications.^23,30^

In this study, we present an innovative, sustainable, and scalable methodology for the creation of two-dimensional sulfur-deficient (2D MoS_2–x_) nanosheets using a deep eutectic solvent (DES) comprised of choline chloride and ethylene glycol. The DES functions as both a dispersing medium for polar and nonpolar species and a passivating and stabilizing agent for the resulting 2D nanosheets with fluorescent behavior. This method is compatible with both hydrothermal synthesis and probe-assisted ultrasonication, offering a versatile and environmentally benign approach for the creation of 2D MoS_2–x_ nanosheets. The structural, chemical, and optical characteristics of the 2D MoS_2–x_ nanosheets are examined using transmission electron microscopy (TEM), X-ray photoelectron spectroscopy (XPS), ultraviolet-visible (UV–vis) spectroscopy, and dynamic light scattering (DLS). These highly fluorescent nanosheets were used as a fluorescence-based nanosensor with a detection limit of 0.40 nM for nifedipine. The designed nanosensor was validated in real-world applications using human urine and pond water, yielding satisfactory recovery results. This approach provides a scalable, sustainable solution for the detection of the calcium channel blocker drug nifedipine via fluorescence-based sensing in real-world samples.

## Experimental Section

### Reagents

Molybdenum­(V) chloride (MoCl_5,_ 99.6%), sodium sulfide (Na_2_S, 98%), ethylene glycol (HOCH_2_CH_2_OH, 99%), choline chloride (CH_3_)_3_N­(Cl)­CH_2_CH_2_OH, >98%), and nifedipine (C_17_H_18_N_2_O_6_, ≥98%) were obtained from Sigma-Aldrich, USA. All reagents were obtained from industrial suppliers and used without further purification.

### Instrumentation

Material creation and characterization were conducted using the following analytical instruments. Ultrasonication was carried out using the UP-800 ultrasonic processor (Chrom Tech, Eagan, MN, USA). UV–visible absorption spectra were measured with an Evolution 201 spectrophotometer (Thermo Scientific, USA). Fluorescence properties were analyzed with an FL-2700 fluorescence spectrophotometer (Hitachi, Japan). Zeta potential and particle size distribution were measured with a dynamic light scattering instrument (EISZ-2000, Otsuka Electronics Co., Ltd., Japan). Surface chemical states were examined using a high-resolution X-ray photoelectron spectrometer (PHI Quantera II, ULVAC-PHI, Japan). High-resolution transmission electron microscopy (HRTEM) images were achieved with a JEOL JEM-3010 analytical scanning transmission electron microscope (Japan). The energy-dispersive X-ray spectroscopy (EDS) system (Link eXL-II) was used to analyze the elemental composition.

### Synthesis of Deep Eutectic Solvent (DES)

DES was synthesized by combining a hydrogen bond acceptor (choline chloride, >98%) and a hydrogen bond donor (ethylene glycol, 99%) in a precise molar ratio (1:2) in a 100 mL glass bottle according to the previously reported method.^14,31^ The mixture was stirred at 80 °C for 1 h using a magnetic stirrer on a hot plate. Stirring continued until a clear, homogeneous liquid was formed. The prepared DES was used directly in subsequent experiments without further purification.

### Synthesis of Fluorescent 2D MoS_2–x_ Nanosheets

Fluorescent 2D MoS_2–x_ nanosheets were synthesized via a DES-assisted hydrothermal method followed by ultrasonication. MoCl_5_ (0.05 M) and Na_2_S (0.1 M) were separately dissolved in 15 mL of DES under stirring for 30 min. The solutions were then combined and stirred at 80 °C for 2 h to ensure homogeneous mixing. The resulting mixture was transferred into a Teflon-lined autoclave and heated at 200 °C for 24 h. After cooling to room temperature, the product was subjected to probe sonication in pulsed mode (3 s on, 1 s off) for 30 min. The precipitate was centrifuged at 6000 rpm for 10 min, washed with deionized (DI) water, and subsequently redispersed in DI water. The dispersion was centrifuged again, and the resulting supernatant was collected for further use. The optical properties of the nanosheets were examined using UV–vis absorption and photoluminescence spectroscopy. A schematic illustration of the synthesis procedure is shown in Figure 1.

**Figure 1:**
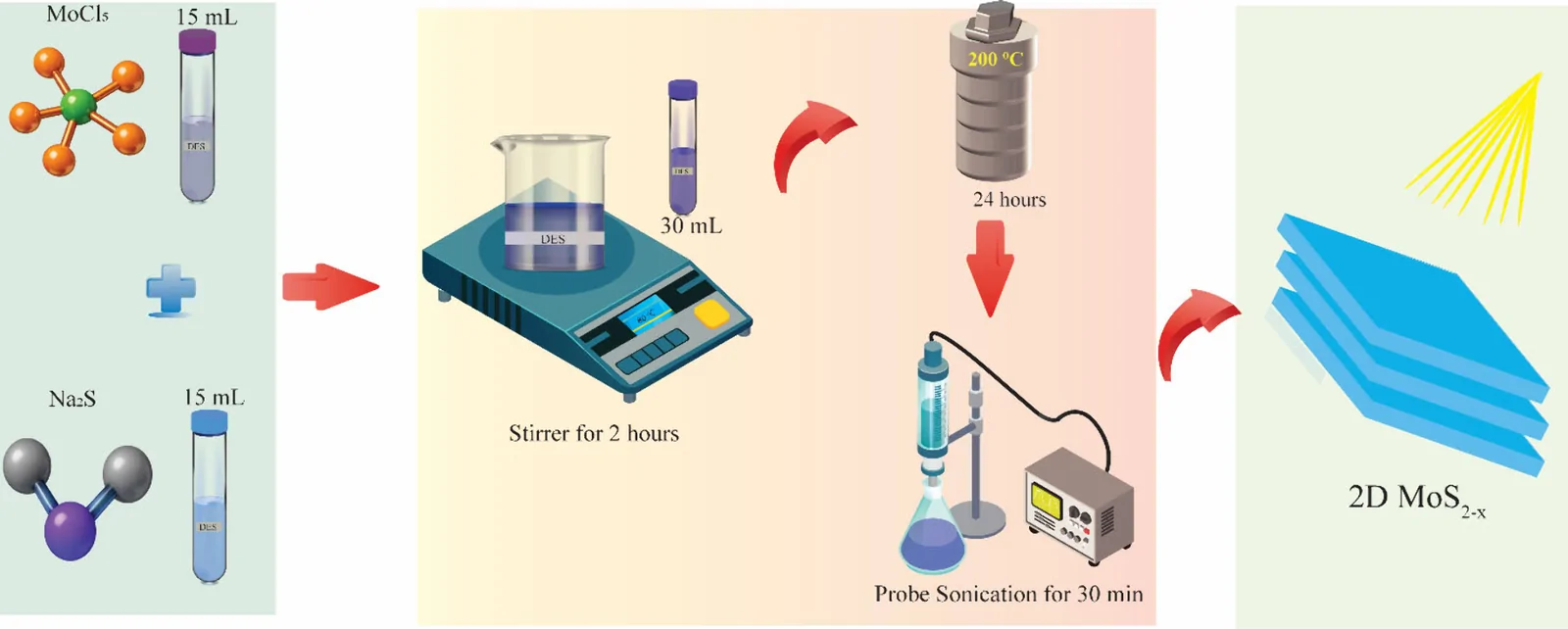
Schematic illustration of the synthesis procedure for fluorescent 2D MoS_2–x_ nanosheets.

### Rapid and Sensitive Detection of NIF in DI Water and Real Samples

To evaluate the fluorescence sensing capability of the synthesized 2D MoS_2–x_ nanosheets, a stock solution of NIF was prepared in dimethyl sulfoxide (DMSO) to ensure complete solubility. Working solutions with varying concentrations of NIF (0–360 nM) were obtained by serial dilution of the stock. For each sensing experiment, 100 μL of the 2D MoS_2–x_ nanosheets suspension was added to the NIF solution and then diluted to a final volume of 1.0 mL using DI water.

To investigate the sensor’s applicability in complex biological and environmental matrices, the protocol was extended to real sample analysis. Human urine and lotus pond water samples were collected from a healthy volunteer and a lotus pond in Kaohsiung, respectively. Both types of samples were centrifuged and subsequently filtered using a Whatman 0.45 μm membrane filter. The samples were spiked with known concentrations of NIF and subjected to the same fluorescence detection procedure. Specifically, 100 μL of the 2D MoS_2–x_ nanosheets dispersion was mixed with each spiked sample and diluted to 1.0 mL with DI water. Fluorescence emission spectra were recorded at an excitation wavelength of 330 nm. All measurements were conducted in triplicate to ensure statistical reliability and reproducibility.

### Selectivity Evaluation via Fluorescence Response

The selectivity of the fluorescent probe was evaluated against a panel of structurally and functionally diverse analytes, including drugs, biomolecules, and potentially interfering metal ions. The analytes tested included nimodipine (NIM), nisoldipine (NSD), ampicillin (AMP), amoxicillin (AMX), chloramphenicol (CAP), dopamine (DA), desipramine (DMI), nicotinamide (NAM), caffeine (CAF), creatine (Cr), acetic acid (AcOH), uric acid (UA), and metal ions such as Zn^2+^, Mg^2+^, and Ca^2+^. All analytes were tested at a concentration of 1 mM. Fluorescence emission spectra were recorded at an excitation wavelength of 330 nm.

## Results and Discussion

### Structural and Compositional Analysis of 2D MoS_2–x_ Nanosheets

Transmission electron microscopy (TEM) and dynamic light scattering (DLS) were applied to study the morphology and structural features of the 2D MoS_2–x_ nanosheets. TEM analysis (Figure 2a,b) revealed the formation of ultrathin, semi-transparent, and elongated 2D nanosheets with relatively large lateral dimensions. The high degree of transparency observed in the TEM images is indicative of the successful exfoliation of bulk MoS_2–x_ into few-layer or monolayer nanosheets. DLS analysis (Figure 2c) further confirmed an average lateral size of approximately 300 nm, consistent with the TEM observations.

**Figure 2:**
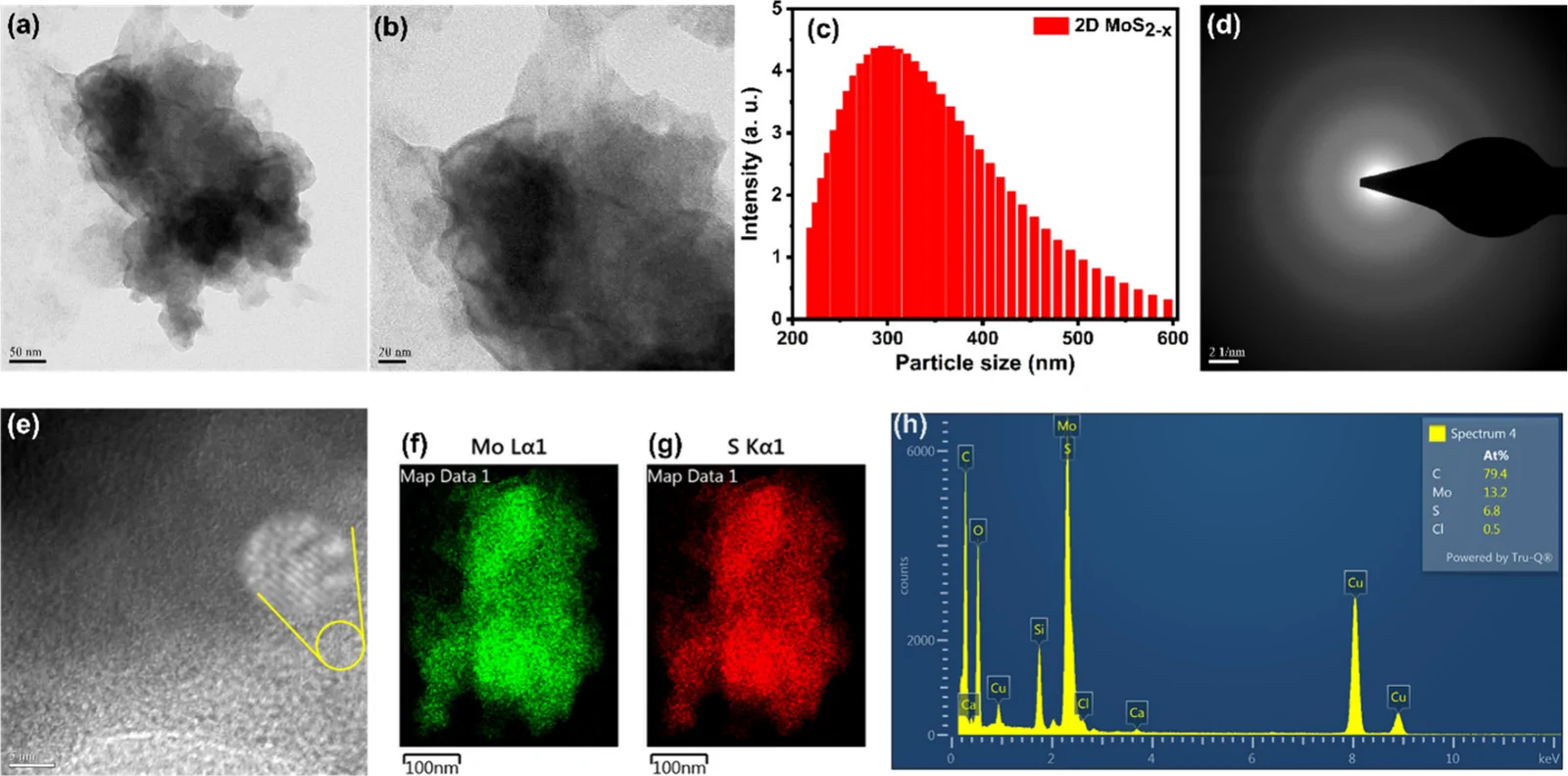
(a,b) TEM images; (c) DLS hydrodynamic size distribution (∼300 nm); (d) SAED pattern; (e) HRTEM image; (f,g) elemental maps showing uniform distribution of Mo (green) and S (red); (h) EDX spectrum confirming elemental composition.

To evaluate the bulk crystallinity of the nanosheets, XRD analysis was performed (Figure 3a). The diffraction pattern exhibits a smooth, monotonically decreasing profile with no discernible Bragg reflections, diffuse humps, or peaks above the statistical noise, confirming that the material is X-ray amorphous and lacks long-range periodic order. However, this featureless XRD profile does not necessarily imply true atomic amorphization. Rather, it arises from a combination of three factors: (i) the extremely small coherent scattering domain sizes (likely below the detection limit of conventional XRD), (ii) the random orientation of these domains within the sheets, and (iii) the presence of substantial structural disorder introduced by sulfur vacancies (MoS_2–x_). Collectively, these effects severely broaden any potential Bragg reflections to the point where they merge into the background, producing an X-ray amorphous-like profile.^32^

**Figure 3:**
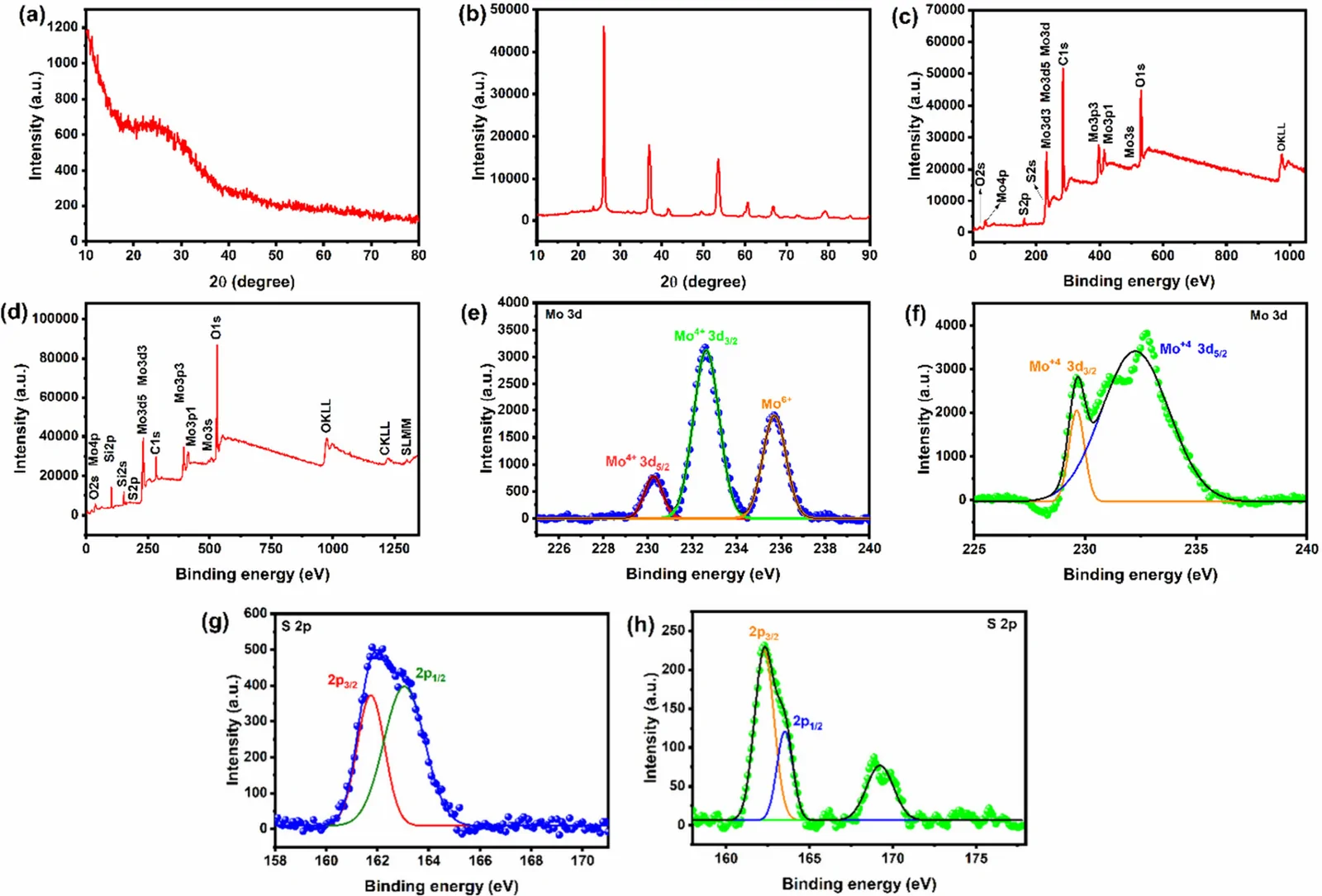
(a) XRD pattern of DES-derived MoS_2–x_; (b) XRD pattern of DI-derived MoS_2_; (c) XPS survey spectrum of DES-derived MoS_2–x_; (d) XPS survey spectrum of DI-derived MoS_2_; (e) high-resolution Mo 3d spectrum of DES-derived MoS_2–x_; (f) high-resolution Mo 3d spectrum of DI-derived MoS_2_; (g) high-resolution S 2p spectrum of DES-derived MoS_2–x_; (h) high-resolution S 2p spectrum of DI-derived MoS_2_.

To complement the bulk XRD results and probe the local structure beyond the XRD detection limit, SAED and HRTEM analyses were performed (Figure 2d,e). The SAED pattern (Figure 2d) exhibits broad diffuse rings rather than discrete spots, which is characteristic of randomly oriented ultrasmall crystalline domains or highly disordered layered structures. Importantly, the d-spacings of these rings are consistent with the interplanar distances of MoS_2_-type structures, indicating the retention of short-range atomic correlations. Consistent with this, HRTEM imaging (Figure 2e) reveals predominantly featureless contrast across most regions; however, closer inspection at higher magnification reveals faint, localized patches of lattice fringes, confirming the presence of ultrasmall nanocrystalline domains embedded within a predominantly disordered matrix.

Taken together, the combined XRD, SAED, and HRTEM evidence indicates that the material is best described as nanocrystalline molybdenum sulfide (MoS_2–x_) with ultrasmall domain sizes and predominantly short-range order, rather than truly amorphous in the strict atomic sense. This observation is consistent with previous reports on poorly crystalline MoS_2_, where X-ray amorphous XRD patterns were accompanied by HRTEM images revealing localized lattice fringes and diffuse SAED rings, attributed to ultrasmall crystallite domains and random stacking of layers.^32^ Importantly, while the material shares chemical similarities with MoS_2_ (Mo–S bonding, sulfur vacancies), it does not possess the long-range layered crystalline structure typical of 2D MoS_2_. Accordingly, comparisons with defect-engineered crystalline MoS_2_ in the literature are therefore most appropriately focused on chemical composition and defect chemistry rather than structural properties. The X-ray amorphous appearance of the XRD pattern is a direct consequence of the ultrasmall coherent domain sizes, random orientation, and sulfur-deficiency-induced disorder, all of which arise from the DES-assisted synthesis and subsequent ultrasonication exfoliation that kinetically arrest long-range crystal growth while preserving local structural correlations.

The elemental distribution within the 2D MoS_2–x_ nanosheets was evaluated using high-angle annular dark field scanning transmission electron microscopy (HAADF-STEM) with EDS mapping. The elemental maps (Figure 2f,g) show a homogeneous distribution of Mo (green) and S (red) across the entire sheet structure, indicating uniform compositional characteristics without significant phase segregation. The corresponding EDS spectrum (Figure 2h) confirmed the presence of Mo and S, revealing a Mo:S atomic ratio of approximately 1.94 (Mo 13.2 at%: S 6.8 at%). This sulfur-deficient stoichiometry is consistent with previous reports showing that the S/Mo ratio decreases with increasing synthesis temperature, leading to the formation of sulfur vacancies. Such sulfur deficiency is a characteristic feature of non-stoichiometric molybdenum sulfide nanosheets and is expected to introduce additional structural disorder within the nanocrystalline domains.^33^

To evaluate the electronic structure and investigate the chemical constituents and oxidation state of the 2D MoS_2–x_ nanosheets, X-ray photoelectron spectroscopy (XPS) was performed. The survey spectrum (Figure 3c) shows that Mo and S are the predominant elements, along with a minor O 1s signal attributed to surface oxidation or adsorbed oxygen species. In the high-resolution Mo 3d spectrum of the DES-derived sample (Figure 3e), two distinct peaks are observed at 230.26 and 232.64 eV, corresponding to Mo 3d_5/2_ and Mo 3d_3/2_ of Mo^4+^, suggesting the dominance of Mo^4+^ in the 2D MoS_2–x_ nanosheets.^34^ A peak at higher binding energy is assigned to the Mo^6+^ species, indicating partial oxidation associated with sulfur vacancy formation. The S 2p spectrum of the DES-derived sample (Figure 3g) shows a typical spin-orbit doublet at 161.74 and 163.04 eV, corresponding to S 2p_3/2_ and S 2p_1/2_ of S^2–^, with no detectable sulfate peaks.

To further confirm the role of DES in sulfur-vacancy formation, a control sample was synthesized under identical hydrothermal conditions without DES (using DI water as solvent). The control sample was characterized alongside the DES-derived material (Figure 3). XRD analysis of the control sample (Figure 3b) exhibited crystalline peaks matching hexagonal MoS_2_ (JCPDS 00-002-0132), with additional peaks attributed to sulfate species. In contrast, the DES-derived sample (Figure 3a) remained X-ray amorphous. XPS survey spectra of both samples are shown in Figures 3c and 3d. The high-resolution Mo 3d spectrum of the control sample (Figure 3f) showed Mo^4+^ predominantly with no detectable Mo^6+^, indicating the absence of significant sulfur vacancies. The S 2p spectrum of the control sample (Figure 3h) revealed both S^2–^ and sulfate peaks, confirming surface oxidation in the absence of DES.

Quantitative XPS analysis was performed by integrating the Mo 3d and S 2p peaks. For the DES-derived sample, the integrated areas of the Mo 3d peaks were 802.71571 (Mo^4+^ 3d_5/2_), 4505.00365 (Mo^4+^ 3d_3/2_), and 2504.63589 (Mo^6+^), yielding a Mo^6+^/Mo^4+^ ratio of 0.472. The S 2p peaks yielded areas of 489.96157 (S^2–^ 2p^3/2^) and 778.04407 (S^2–^ 2p_1/2_), with no detectable sulfate. After applying the appropriate relative sensitivity factors, the S/Mo atomic ratio was determined to be 0.775, significantly lower than the stoichiometric value of 2.0, confirming severe sulfur deficiency. In contrast, the control sample exhibited a Mo^6+^/Mo^4+^ ratio of 0 (no detectable Mo^+6^), an S/Mo atomic ratio of 0.199, and an S^2–^/Sulfate ratio of 3.22, confirming the presence of sulfate and fewer sulfur vacancies.

These results conclusively demonstrate that DES plays a dual role: (i) introducing sulfur vacancies by disrupting long-range crystallinity (as evidenced by the higher Mo^6+^/Mo^4+^ ratio, lower S/Mo ratio, and amorphous XRD pattern in the DES-derived sample), and (ii) passivating the surface to prevent sulfate formation (as evidenced by the absence of sulfate peaks in the DES-derived sample, whereas the control sample showed clear sulfate peaks). These findings are consistent with defect-engineered molybdenum sulfide reported previously.^34,35^

### Optical Characterization via PL and UV–Vis Spectroscopy

The optical properties of the 2D MoS_2–x_ nanosheets were investigated using UV–visible absorption and photoluminescence (PL) spectroscopy. The UV-visible absorption spectrum of the nanosheets (Figure 4a) shows a peak in the range of 236–263 nm, along with a broadened absorption extending up to 400 nm, confirming the successful synthesis of the 2D MoS_2–x_ nanosheets. Figure 4b shows the PL excitation (330 nm) and emission (420 nm) spectra. Excitation-dependent emission optimization (Figure 4c) revealed maximum intensity at 330 nm. As shown in Figure 4d, the results demonstrate that the 2D MoS_2–x_ nanosheets function as a fluorescence-based nanosensor for NIF detection, showing significant quenching even at very low concentrations. Additionally, a slight red shift in the fluorescence emission of 2D MoS_2–x_ nanosheets was observed upon the addition of NIF. This shift is consistent across the entire concentration range (0.6–360 nM) and does not exhibit concentration-dependent behavior, suggesting ground-state complex formation between 2D MoS_2–x_ nanosheets and NIF. Consequently, this constant shift is not suitable for ratiometric sensing; therefore, the intensity-based quenching response remains the preferred analytical approach. The spectral shift is attributed to specific interactions between the 2D MoS_2–x_ surface and the −NO_2_ functional groups of NIF, where dipole–dipole interactions or electron transfer perturb the local electronic environment, resulting in a bathochromic shift in emission.^36^ To rigorously establish the origin of the fluorescence and exclude the possibility of fluorescent DES-derived, organic, or carbonaceous species, comprehensive control experiments were performed under identical hydrothermal conditions (200°C, 24 h). Photoluminescence spectra of all control samples were recorded at λ_ex_ = 330 nm (Supporting Information, Figure S1). DES alone showed no fluorescence, confirming that the DES does not produce emissive species. Na_2_S and MoCl_5_ in water and aqueous synthesis without DES (MoCl_5_ + Na_2_S + H_2_O) showed no fluorescence. These control experiments collectively confirm that the strong blue fluorescence (λ_em_ = 420 nm) originates solely from the sulfur vacancy-mediated defect states in the 2D MoS_2–x_ nanosheets.

**Figure 4:**
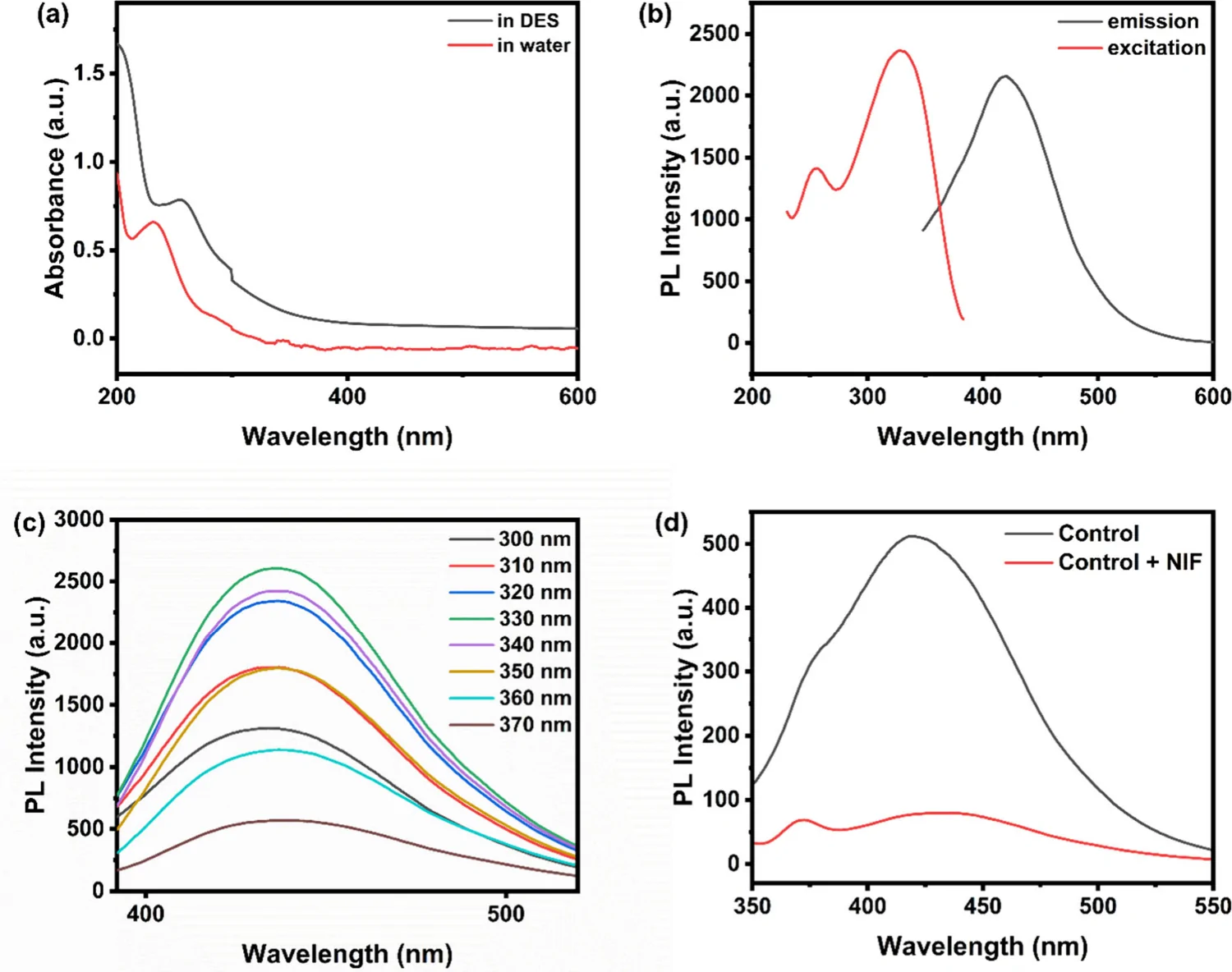
(a) UV–vis absorption spectra of DES-derived MoS_2–x_ and DI water-derived MoS_2_; (b) PL excitation and emission spectra of DES-derived MoS_2–x_ nanosheets; (c) PL emission spectra recorded at different excitation wavelengths; (d) PL spectra of DES-derived MoS_2–x_ nanosheets before and after NIF addition.

### Fluorescence Optimization of 2D MoS_2–x_ Nanosheets

The practical performance of the 2D MoS_2–x_ was evaluated under various environmental conditions. The temperature-dependent photoluminescence behavior of the material was systematically evaluated over the range of 30–80 °C. As shown in Figure 5a, the material exhibited high thermal stability, with minimal variation in emission intensity across the studied range. The fluorescence intensity reached a maximum at 30 °C, followed by a gradual decline with increasing temperature up to 80 °C. These observations suggest that the material undergoes limited thermal quenching, with 30 °C being the optimal temperature for maximal photoluminescence efficiency.

**Figure 5:**
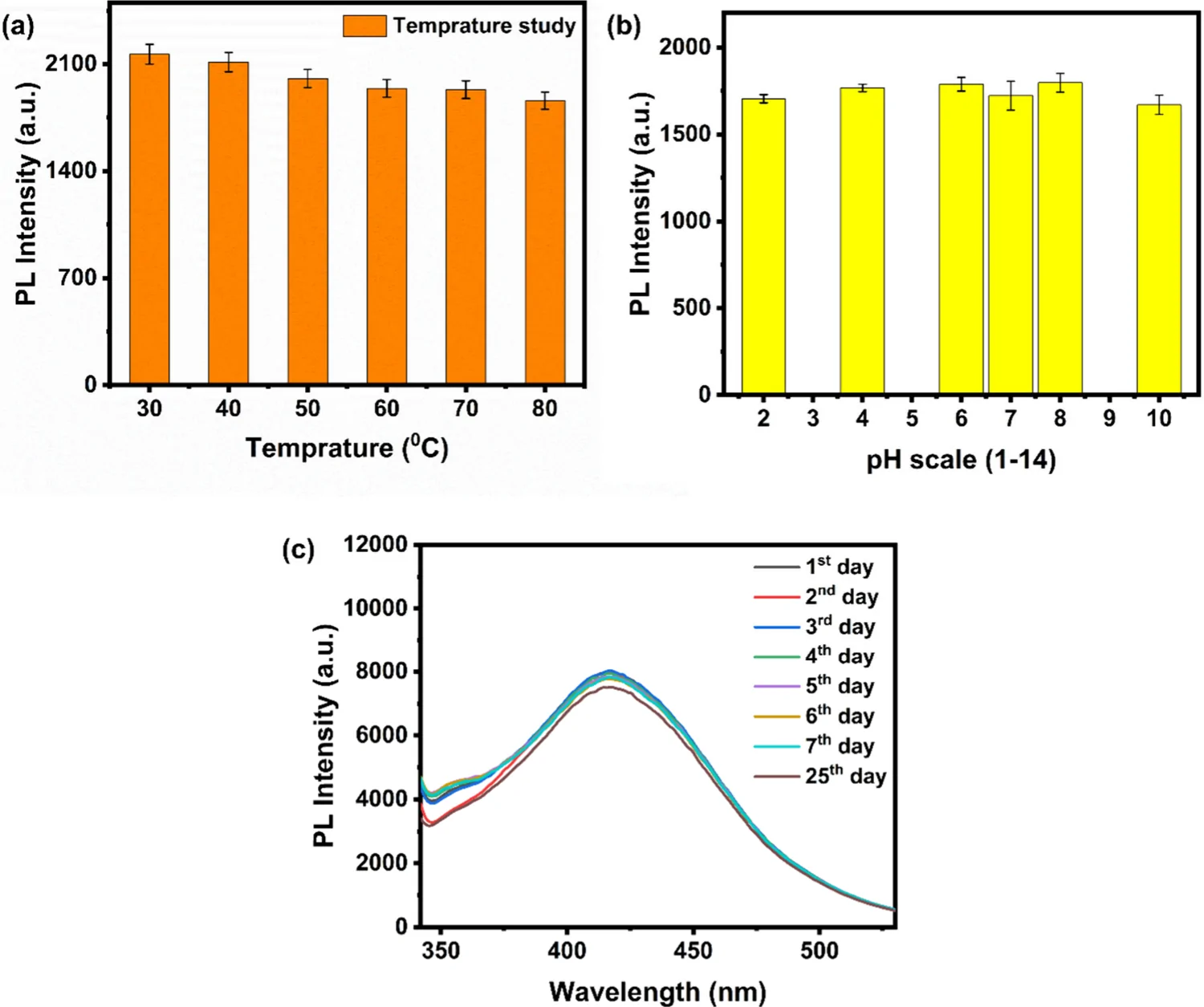
(a) Temperature-dependent fluorescence of 2D MoS_2–x_ nanosheets (30–80 ^0^C); (b) pH-dependent fluorescence (pH 2–10); (c) time-dependent fluorescence stability (1–25 days).

To evaluate the applicability of the nanosheets in complex biological and environmental matrices, their fluorescence response was examined across a broad pH range (2–10). As depicted in Figure 5b, the nanosheets maintained a stable fluorescence signal over the tested pH values, with optimal stability observed at pH 6 and 8. These results indicate that the designed nanosheets exhibit favorable photostability and pH tolerance, highlighting their suitability for practical applications in complex sample environments.

Finally, the photoluminescence stability of the nanosensor was assessed over 25 days under ambient conditions, as illustrated in Figure 5c. Fluorescence measurements were recorded. While minor fluctuations in intensity were observed, no significant trends indicating degradation or enhancement were detected. The emission profile remained consistent throughout the monitoring period, demonstrating that the material maintains stable photoluminescent properties over time. This stability is essential for applications requiring long-term performance and optical reliability.

### Detection of NIF Using the 2D MoS_2–x_ Nanosheets

Following fluorescence optimization, the 2D MoS_2–x_ nanosheets were employed as a sensing platform for NIF detection, a therapeutically significant compound on the World Health Organization’s (WHO) List of Essential Medicines.^37^ The sensitivity of the developed probe was systematically evaluated by monitoring its fluorescence response upon addition of varying concentrations of NIF (0.60–360 nM) while maintaining a constant concentration of 2D MoS_2–x_ nanosheets. A red shift (∼8 nm) in the fluorescence emission maximum from approximately 420 nm to approximately 428 nm was observed upon the introduction of NIF. The shift remained nearly constant across the investigated concentration range, indicating a strong interaction between the probe and the analyte and suggesting that the sensing behavior is primarily driven by photoinduced electron transfer from the nanosheets to NIF.^38^ The corresponding fluorescence intensity changes are shown in Figure 6a. Increasing the NIF concentration induced fluorescence quenching, resulting in a progressive reduction in the intense blue emission of the 2D MoS_2–x_ nanosheets. A highly linear fluorescence-quenching response was obtained for NIF, with a correlation coefficient (R^2^) of 0.993. The LOD was calculated as 0.40 nM from the standard deviation of the blank measurements and the slope of the linear calibration curve**.** The residual plot for the calibration curve (Supporting Information, Figure S2) shows randomly scattered residuals around the zero line with no obvious trend, confirming the validity of the linear regression model. The quenching ratio, calculated as (*F*_o_/F), was plotted against the NIF concentration, as shown in Figure 6b.

**Figure 6:**
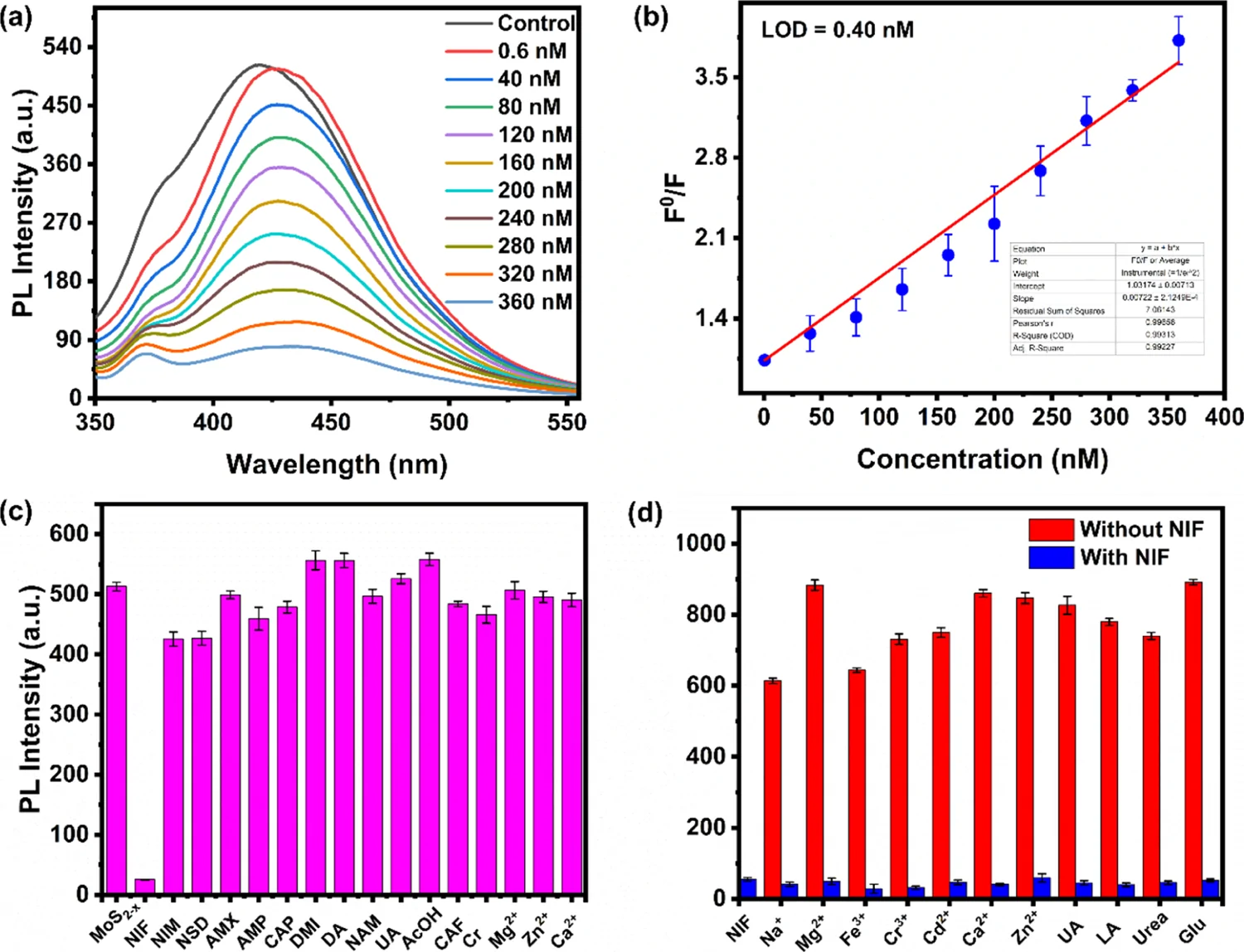
Fluorescence response of the 2D MoS_2–x_ nanosheets toward NIF: (a) quenching of fluorescence intensity with increasing NIF concentration; (b) linear regression analysis illustrating the sensor’s sensitivity to NIF; (c) selectivity of the probe toward NIF over various interferents; (d) anti-interference study in the presence of common coexisting species.

The selectivity of the probe was first assessed against various structurally and functionally diverse analytes, including nimodipine (NIM), nisoldipine (NSD), ampicillin (AMP), amoxicillin (AMX), chloramphenicol (CAP), dopamine (DA), desipramine (DMI), nicotinamide (NAM), caffeine (CAF), creatine (Cr), acetic acid (AcOH), uric acid (UA), as well as metal ions (Zn^2+^, Mg^2+^, Ca^2+^), all at 1 mM. As shown in Figure 6c and Supporting Information, Figure S3, NIF exhibited a strong quenching effect upon interaction with the 2D MoS_2–x_ nanosheets, whereas the tested interferents showed a negligible fluorescence response, confirming the high selectivity of the developed nanosensor toward NIF.

The anti-interference capability of the developed probe was evaluated in the presence of common metal ions (Ca^2+^, Fe^3+^, Mg^2+^, Na^+^, Zn^2+^, Cr^3+^, Cd^2+^) and biomolecules (uric acid, lactic acid, urea, glucose) at 1 mM. As shown in Figure 6d, these interferents showed negligible impact on the fluorescence response of the 2D MoS_2–x_ nanosheets, confirming the probe’s high selectivity under complex conditions. To assess the sensor’s performance in complex matrices, a mixture of interferents (10, 50, and 100 μM) was spiked into urine and lotus pond water samples containing 1 μM NIF. The fluorescence response was recorded in the presence and absence of NIF (Supporting Information, Figures S4 and S5). The results demonstrate that the interferent mixture did not significantly affect the NIF-induced quenching, confirming the high selectivity of the nanosensor in real-world samples

The analytical performance of the 2D MoS_2–x_ nanosensor for NIF detection was systematically compared with previously reported techniques, including electrochemical (EC), cyclic voltammetry (CV), photoelectrochemical (PEC), and differential pulse voltammetry (DPV) methods (Table 1), as well as optical sensing platforms (Table 2). The 2D MoS_2–x_ nanosheets exhibited superior sensitivity, achieving an extremely low limit of detection (LOD) compared to conventional techniques. Although EC-based techniques offer rapid and cost-effective analysis, their selectivity is often compromised in complex matrices due to overlapping redox potentials.^39^ In contrast, fluorescence-based systems, such as the 2D MoS_2–x_ nanosheets, provide improved sensitivity and selectivity, making them more suitable for real-sample applications.^40^ The combination of high sensitivity, low LOD, fast response, and cost-efficiency highlights the capability of the 2D MoS_2–x_ nanosheets as a robust and practical sensing platform for NIF detection in complex environments.

**Table 1:** Analytical Parameters Obtained in This Study Compared with Those Reported in the Literature

material	technique (nM)	linear range (nM)	LOD	ref
MnNiO_3_/DCE	EC	100–66100	19	^41^
Bi_3_NbO7	EC	49–1502.89	9600	^7^
AgNPs	DPV	800–60000	720	^5^
BaSnO_3_/GCE	DPV	10–697650	16.8	^42^
MgO–SPEs	DPV	200–104410	32	^43^
AP-BDDE	DPV	3980–107000	612	^44^
MWNT-COOH/PANI/ITO	CV	1000–100000	1600	^45^
CuTAPc/ITO	PEC	250–18000	15000	^46^
MWCNTs/CD	DPV	70–15000	25	^47^
HMDmE	DPV	200–20000	120	^48^
AlPc-CC POP	PDV	40–50000	27.3	^1^
βCD-CNT-PE	PDV	48–20000	15	^49^
2D MoS_2–x_ nanosheets	FL	0.6–360	0.40	this work

**Table 2:** Analytical Parameters Obtained in This Work Compared with Those Reported in Fluorescence-Based Studies

material	technique (nM)	linear range (nM)	LOD	ref
MIP@SiC-dots	FL	280–3400	76	^50^
2D-TTO nanosheet	FL	50–1000	7.2	^8^
2D MoS_2–x_ nanosheets	FL	0.6–360	0.40	this work

The practical reliability of the 2D MoS_2–x_ nanosensor was further evaluated in terms of response time, precision, stability, matrix effects, and solvent tolerance. The sensor exhibited an immediate response to NIF, reaching equilibrium within <1 min (Supporting Information, Figure S6), indicating rapid analyte detection. Intra-day and inter-day precision were assessed by measuring the fluorescence response at different time intervals on the same day and over consecutive days, respectively; the fluorescence intensity remained consistent across all measurements (Supporting Information, Figures S7 and S8), confirming excellent reproducibility of the sensor. The sensor also showed good stability over the tested period, with no significant loss in fluorescence intensity. The tolerance to DMSO content (1–5%, v/v) was evaluated, and the sensor performance remained unaffected (Supporting Information, Figure S9). Matrix-matched calibration in urine and pond water (Table 3) confirmed the reliability of the sensor in complex real samples. These results collectively demonstrate the practical reliability of the developed nanosensor for NIF detection in real-world samples.

**Table 3:** Recovery Analysis of Nifedipine in Real Samples

biological sample (human urine)			
spiked (nM)	found (nM)	recovery %	RSD %
0.6	0.61	99.83	0.78
40	37.81	94.53	1.97
80	75.65	94.56	1.35
120	108.01	90.01	2.40
160	135.70	84.81	0.98

environmental sample (lotus pond water)			
spiked (nM)	found (nM)	recovery %	RSD %
0.6	0.59	99.48	1.53
40	36.74	91.84	2.27
80	73.79	92.23	1.53
120	108.32	90.26	2.57
160	134.09	83.80	2.39

### Fluorescence Quenching Mechanism of the Nanosensor

The fluorescence quenching mechanism of the 2D MoS_2–x_ nanosensor upon NIF addition was systematically investigated using a combination of spectroscopic techniques, including time-resolved photoluminescence (TRPL), temperature-dependent Stern–Volmer analysis, UV–vis absorption spectroscopy, FTIR, zeta potential measurements, and absorbance-corrected fluorescence analysis. The quenching proceeds through a combination of static quenching, inner filter effect (IFE), photoinduced electron transfer (PET), and hydrogen bonding stabilization.

Ground-state complex formation between the 2D MoS_2–x_ nanosheets and NIF was confirmed by UV–vis absorption spectroscopy. The pristine nanosheets exhibited a distinct absorption maximum at 256 nm, corresponding to their intrinsic electronic transition, while NIF displayed characteristic absorption bands at 207 and 237 nm, along with a broad feature extending from 305 to 426 nm. Upon interaction with the nanosheets, two new absorption bands appeared at 251 and 318 nm, indicating the formation of a ground-state complex through strong electronic interactions and possible charge transfer between the analyte and the nanosheet surface (Figure 7a).^51,52^ Static quenching was further confirmed by TRPL measurements, where the fluorescence lifetime increased from 13.9 to 15.87 ns (+14.2%) upon NIF addition, consistent with the formation of a non-fluorescent ground-state complex as shown in Figure 7b. Additionally, temperature-dependent Stern–Volmer analysis (Supporting Information, Figure S10) revealed a decrease in the Stern–Volmer constant (K_SV_) with increasing temperature (from 0.01663 to 0.0100 nM^–1^), which is characteristic of static quenching, as higher temperatures destabilize the ground-state complex.^53^ Zeta potential measurements further supported electrostatic interaction between the nanosheets and NIF; the pristine 2D MoS_2–x_ nanosheets exhibited a negatively charged surface (−13.16 mV), which shifted to −7.92 mV upon NIF addition, indicating meaningful electrostatic association between the negatively charged nanosheet surface and the drug molecules (Figure 7c,d).

**Figure 7:**
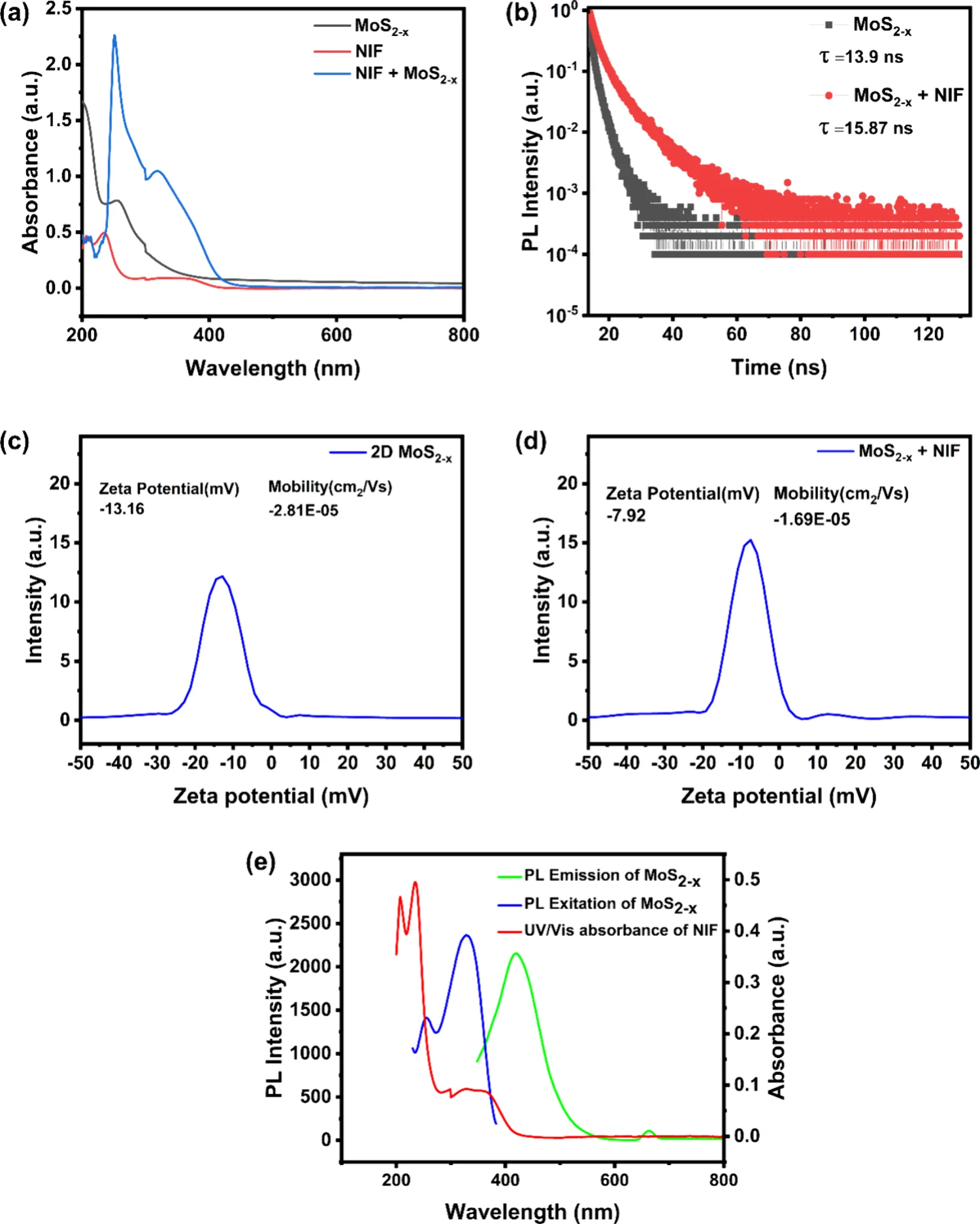
(a) UV–vis absorption spectra of 2D MoS_2–x_, NIF, and their mixture; (b) time-resolved photoluminescence (TRPL) decay profiles of 2D MoS_2–x_ in the absence and presence of NIF; (c) zeta potential of pristine 2D MoS_2–x_ nanosheets; (d) zeta potential of 2D MoS_2–x_ after NIF addition; (e) photoluminescence excitation and emission spectrum of 2D MoS_2–x_. And the UV–vis absorption spectrum of NIF.

The inner filter effect (IFE) was evaluated by examining the spectral overlap between the UV–vis absorption spectrum of NIF and the excitation and emission spectra of the 2D MoS_2–x_ nanosheets (Figure 7e). The significant overlap of the absorbance spectrum of NIF with the excitation spectrum of the nanosheets suggested a potential contribution of IFE to the observed quenching.^54^ To quantify this contribution, fluorescence intensities were corrected using the Lakowicz equation:

$$F_{\mathrm{corrected}}=F_{\mathrm{observed}}\times 10^{A_{\mathrm{ex}}+A_{\mathrm{em}}/2}$$

Where *A*_ex_ and *A*_em_ are the absorbances at the excitation (330 nm) and emission (420 nm) wavelengths, respectively.^54^ Since the absorbance of NIF at the emission wavelength (420 nm) was negligible, the correction was simplified to *F*_corr_ = *F*_abs_ × 10^(*A*ex/2)^. Over the concentration range of 40–360 nM (where absorbance was measurable for IFE correction), the apparent Stern–Volmer slope (K_SV_ = 0.0084 nM^–1^) decreased to 0.0065 nM^–1^ after IFE correction, corresponding to a 22.6% reduction (Supporting Information, Figure S11). This confirms that IFE contributes only partially to the overall quenching, while other mechanisms dominate.

Photoinduced electron transfer (PET) was identified as the dominant quenching mechanism. The molecular structure of NIF contains electron-withdrawing groups, including a nitro group (−NO_2_) and ester moieties, which render the molecule electron-deficient. Upon photoexcitation at 330 nm, electrons are transferred from the electron-rich 2D MoS_2–x_ nanosheets (donor) to the electron-deficient nitro group of NIF (acceptor), reducing it to a hydroxylamine intermediate.^8^ PET was confirmed by FTIR spectroscopy, which showed diminished NO_2_ stretching peaks (1520–1530 and 1340–1350 cm^–1^) and the appearance of a new N–H bending peak at 1645 cm^–1^, and a broad O–H/N–H stretching band at 3323 cm^–1^ (Supporting Information, Figure S12). Additionally, bandgap analysis using Tauc plots revealed a narrowing of the optical bandgap from 2.62 to 2.13 eV (ΔEg = 0.49 eV) upon NIF interaction (Supporting Information, Figures S13, S14), consistent with charge transfer-induced electronic perturbation. The synergistic effects of PET and IFE have been demonstrated as a combined quenching mechanism in fluorescence sensing systems.^55^ PET contributes approximately 77.4% of the total quenching, establishing it as the dominant mechanism, while IFE accounts for the remaining 22.6%.

Finally, hydrogen bonding stabilization of the charge-transfer complex was evidenced by FTIR spectroscopy. The hydroxylamine intermediate formed upon nitro reduction engages in hydrogen bonding with the ester carbonyl groups of NIF, as confirmed by the appearance of a new O–H/N–H stretching band at 3323 cm^–1^ and the diminished C=O stretching vibration at 1680 cm^–1^. This hydrogen bonding interaction stabilizes the charge-transfer complex, facilitating efficient electron transfer and enhancing the overall quenching efficiency.

In summary, the fluorescence quenching of the 2D MoS_2–x_ nanosensor by NIF proceeds through a multi-step mechanism (Figure 8): (i) ground-state complex formation (static quenching, prerequisite), (ii) inner filter effect (minor contribution, ∼22.6%), (iii) photoinduced electron transfer from the nanosheets to the electron-deficient nitro group of NIF (dominant contribution, ∼77.4%), and (iv) hydrogen bonding stabilization of the charge-transfer complex. This combined mechanism accounts for the high sensitivity and selectivity of the developed nanosensor toward NIF detection.

**Figure 8:**
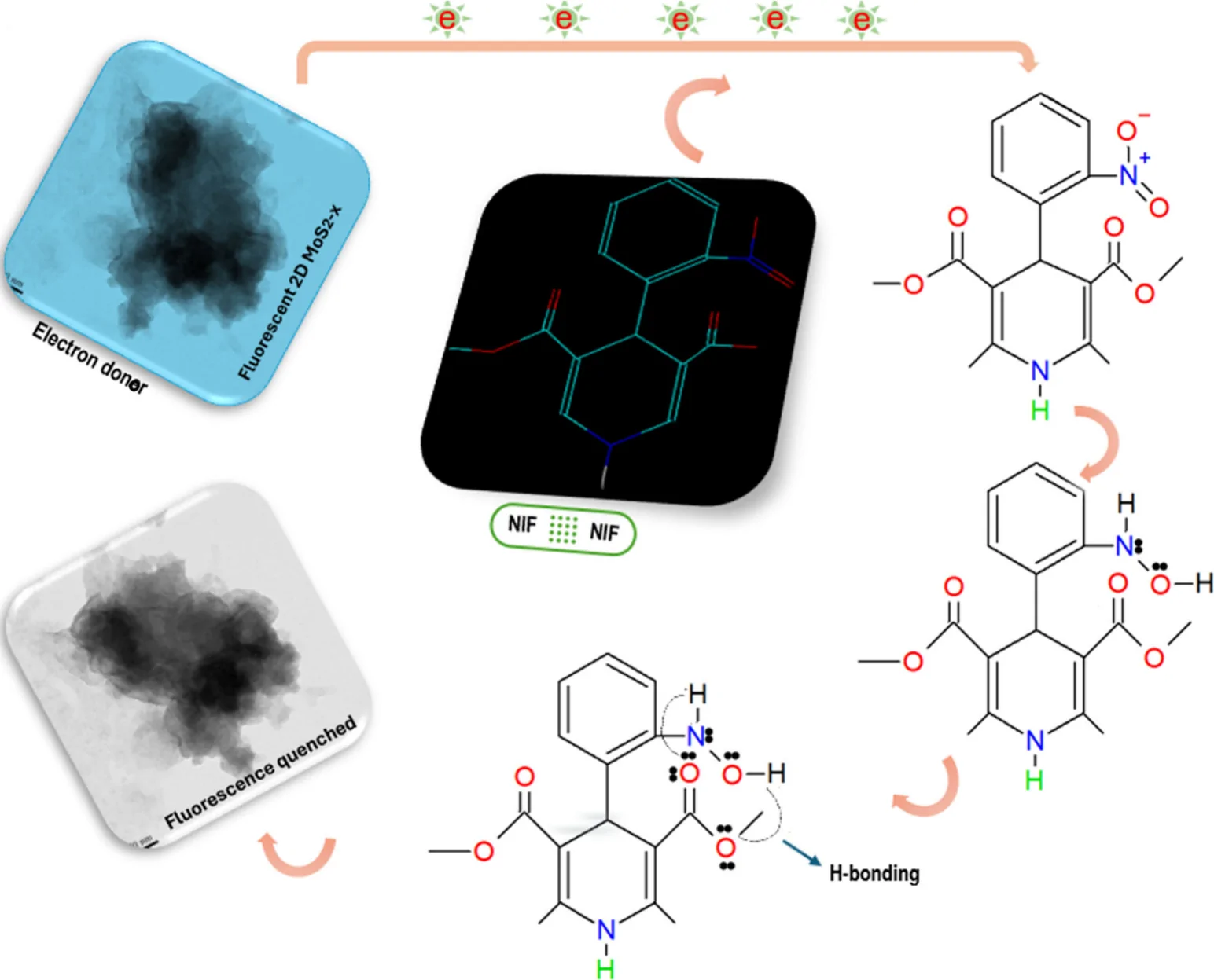
Proposed fluorescence quenching mechanism of 2D MoS_2–x_ upon interaction with NIF. The observed decrease in PL intensity is attributed to electron transfer from 2D MoS_2–x_ nanosheets to the surface of NIF.

### Nifedipine Tracings in Urine and Lotus Pond Water

Following successful detection of NIF in DI water, the applicability of the nanosensor was further evaluated in real matrices, including human urine obtained from healthy donors and water samples collected from Kaohsiung Lotus Pond. The samples were spiked with different concentrations of NIF (0.6–160 nM) and analysed using the developed fluorescence platform. The PL intensity exhibited concentration-dependent quenching, with progressively decreased emission upon increasing NIF concentration (Supporting Information, Figure S15a,b). A linear correlation between the quenching factor and NIF concentration was established based on the Stern–Volmer model, affording correlation coefficients of R^2^ = 0.999 for urine and R^2^ = 0.994 for lotus pond water (Supporting Information, Figure S15c,d).

The calculated recovery values in Table 3 ranged from 84.8% to 99.8% in urine and 83.8–99.5% in pond water, with RSD values below 3% in all cases. A systematic decrease in recovery was observed at higher spiked concentrations (160 nM), dropping to approximately 84–92%, which is likely attributed to matrix-induced suppression from proteins, salts, and dissolved organic matter, as well as non-specific adsorption. Similar matrix effects have been reported in fluorescence sensing, where proteins bind to analytes via hydrogen bonds and van der Waals forces, leading to signal loss.^56^ A control experiment confirmed that DMSO (1–5%) did not affect the quenching response (Supporting Information, Figure S9), ruling out solvent interference. While recoveries >80% with RSD < 3% are acceptable for fluorescence-based sensing in complex matrices.^40^ These results demonstrate the reliable performance of the proposed fluorescence probe in complex biological and environmental samples.

### Green Chemistry Assessment

To evaluate the environmental impact of the DES-based synthesis, a mung bean (Vigna radiata) seed germination test was performed. Seeds were germinated in both tap water (control) and the reaction residue (waste obtained after centrifugation of the 2D MoS_2–x_ synthesis) in the dark. Germination in tap water began on day 2, while in the reaction residue it began on day 3. After 5 days of growth, both sets showed successful germination with comparable growth rates (Supporting Information, Figure S16). The one-day delay is likely due to the high ionic strength of the DES-based waste, but successful germination confirms the non-phytotoxic nature of the synthesis waste. While the core DES components (choline chloride and ethylene glycol) are inherently biodegradable and non-toxic, we acknowledge that the 24 h hydrothermal step at 200°C introduces a high energy demand. This thermal input is mandatory to secure the specific sulfur vacancy density and 2D morphology. However, from a broader sustainability perspective, this localized energy consumption is offset by the elimination of toxic precursors and the subsequent energy-intensive waste treatment processes required by traditional methods. DMSO was used for NIF stock preparation due to the limited aqueous solubility of nifedipine (final concentration <1 % v/v. To further optimize this energy footprint, future studies will explore alternative, lower-temperature DES systems, recycling protocols, and standardized greenness metrics.

## Conclusion

This study demonstrates a green and efficient strategy for synthesizing fluorescent sulfur-deficient 2D MoS_2–x_ nanosheets using a DES, which acts both as a solvent and a surface passivating agent. The resulting 2D MoS_2–x_ nanosheets exhibit strong photoluminescence *λ*_max_ 420 nm under 330 nm excitation and serve as a sensitive and selective probe for the detection of NIF. The sensor achieved a low detection limit of 0.40 nM and a broad linear range (0.6–360 nM), with excellent selectivity over related compounds and metal ions. A rapid response and 8 nm red-shift in the emission, even at low analyte concentrations, indicate strong interaction between 2D MoS_2–x_ nanosheets and NIF. Mechanistic studies support electron transfer and IFE as the dominant quenching pathways. The successful application of the sensor in environmental and biological matrices demonstrates its potential for real-world pharmaceutical monitoring. This work offers a sustainable and effective platform for trace-level sensing based on DES-assisted and 2D nanomaterials.

## Supplementary Material



## Data Availability

The data supporting the findings of this study are available within the article and its Supporting Information. Additional data that support the findings of this study are available from the corresponding author upon reasonable request.

## Notes

Additional experimental details; fluorescence control experiments; residual plot for calibration curve; comparative quenching efficiencies of NIF, structural analogues, pharmaceutical drugs, and metal ions; combined interference studies in urine and pond water; response time analysis; intra-day and inter-day precision; DMSO tolerance; temperature-dependent Stern–Volmer analysis; IFE-corrected Stern–Volmer plots; FTIR spectra; Tauc plots for bandgap determination; real sample analysis in urine and pond water; mung bean seed germination test; statistical validation and LOD calculation; calibration curve parameters; blank replicates and LOD calculation; and quenching efficiency analysis (PDF)
