# Supplementary material for: DES-Based Green Synthesis of 2D Fluorescent Molybdenum Disulfide for Highly Sensitive and Selective Detection of Nifedipine
Source: Anal Chem. 2026 Oct 1;98(41):29445–58. doi: 10.1021/acs.analchem.6c02269 (PMC13689398; doi:10.1021/acs.analchem.6c02269)
Supplement: Supplementary file 1 [file ac6c02269_si_001.pdf]

Supporting Information

DES-Based Green Synthesis of 2D Fluorescent Molybdenum Disulfide for Highly Sensitive and Selective Detection of Nifedipine

Rafee Ullah[1], Shams Ur Rehman[1], Adnan Khan[1], Hui-Fen Wu*[1, 2, 3,4,5,6]

[1]Department of Chemistry, National Sun Yat-Sen University, Kaohsiung, 70, Lien-Hai Road, Kaohsiung, 80424, Taiwan

[2]School of Pharmacy, College of Pharmacy, Kaohsiung Medical University, Kaohsiung, 807, Taiwan

[3]School of Medicine, College of Medicine, National Sun Yat-Sen University, Kaohsiung, 80424, Taiwan

[4]Institute of Medical Science and Technology, National Sun Yat-Sen University, Kaohsiung, 80424, Taiwan

[5]Institute of Precision Medicine, National Sun Yat-Sen University, Kaohsiung, 80424, Taiwan

[6]Institute of Bio Pharmaceutical Science, National Sun Yat-Sen University, Kaohsiung, 80424, Taiwan

*Corresponding author, Prof Hui-Fen Wu (Department of Chemistry, National Sun Yat-Sen University)

Phone: +886-7-5252000-3955; Fax: +886-7-5253909

Email: hwu@faculty.nsysu.edu.tw (Prof H.-F. Wu, Department of Chemistry, National Sun Yat-Sen University)

Table of Contents:

Figure S1. PL spectra of control experiments (DES alone, $MoCl_5$ in water, Na_2S in water, aqueous synthesis without DES, and 2D MoS_{2-x} nanosheets)..S4

Figure S2. Residual plot for the calibration curve of NIF detection using 2D MoS_{2-x} nanosheets… S4

Figure S3. Comparative quenching efficiencies of NIF, structural analogues, pharmaceutical drugs, and metal ions. S5

Figure S4. Fluorescence response of 2D MoS_{2-x} nanosensors in urine spiked with interferents (10–100 µM) in the presence and absence of NIF (1 µM). S5

Figure S5. Fluorescence response of 2D MoS_{2-x} nanosensors in lotus pond water spiked with interferents (10–100 µM) in the presence and absence of NIF (1 µM). S6

Figure S6. Response time analysis of the 2D MoS_{2-x} nanosensor upon addition of NIF (150 nM)..S6

Figure S7. Intra-day fluorescence response of 2D MoS_{2-x} nanosheets upon NIF addition measured at different time intervals on the same day...S7

Figure S8. Inter-day fluorescence response of 2D MoS_{2-x} nanosheets upon NIF addition measured over different days. S7

Figure S9. Effect of DMSO concentration (1–5%, v/v) on the fluorescence intensity of 2D MoS_{2-x} nanosheets..S8

Figure S10. Temperature-dependent Stern-Volmer plot for the 2D MoS_{2-x} nanosheet-NIF system… S8

Figure S11. Stern-Volmer plots of 2D MoS_{2-x} before (black) and after (red) quantitative IFE correction… S9

Figure S12. FTIR spectra of pristine NIF and 2D MoS_{2-x} nanosheets after interaction with NIF… S9

Figure S13. Tauc plot for indirect bandgap determination of pristine 2D MoS_{2-x} nanosheets (bandgap = 2.62 eV). S10

Figure S14. Tauc plot for indirect bandgap determination of 2D MoS_{2-x} after interaction with NIF (bandgap = 2.13 eV)...S10

Figure S15. PL response of the 2D MoS_{2-x} nanosensor for real sample analysis: (a) urine; (b) pond water; (c) urine calibration plot; (d) pond water calibration plot…...............................S11

Figure S16. Mung bean seed germination test in tap water (control) and reaction residue after 5 days...S11

Calculation Section. Statistical Validation, Limit of Detection (LOD) Calculation, Table S1, Table S2, and Quenching Efficiency Calculation.……………..………………………S11-S13

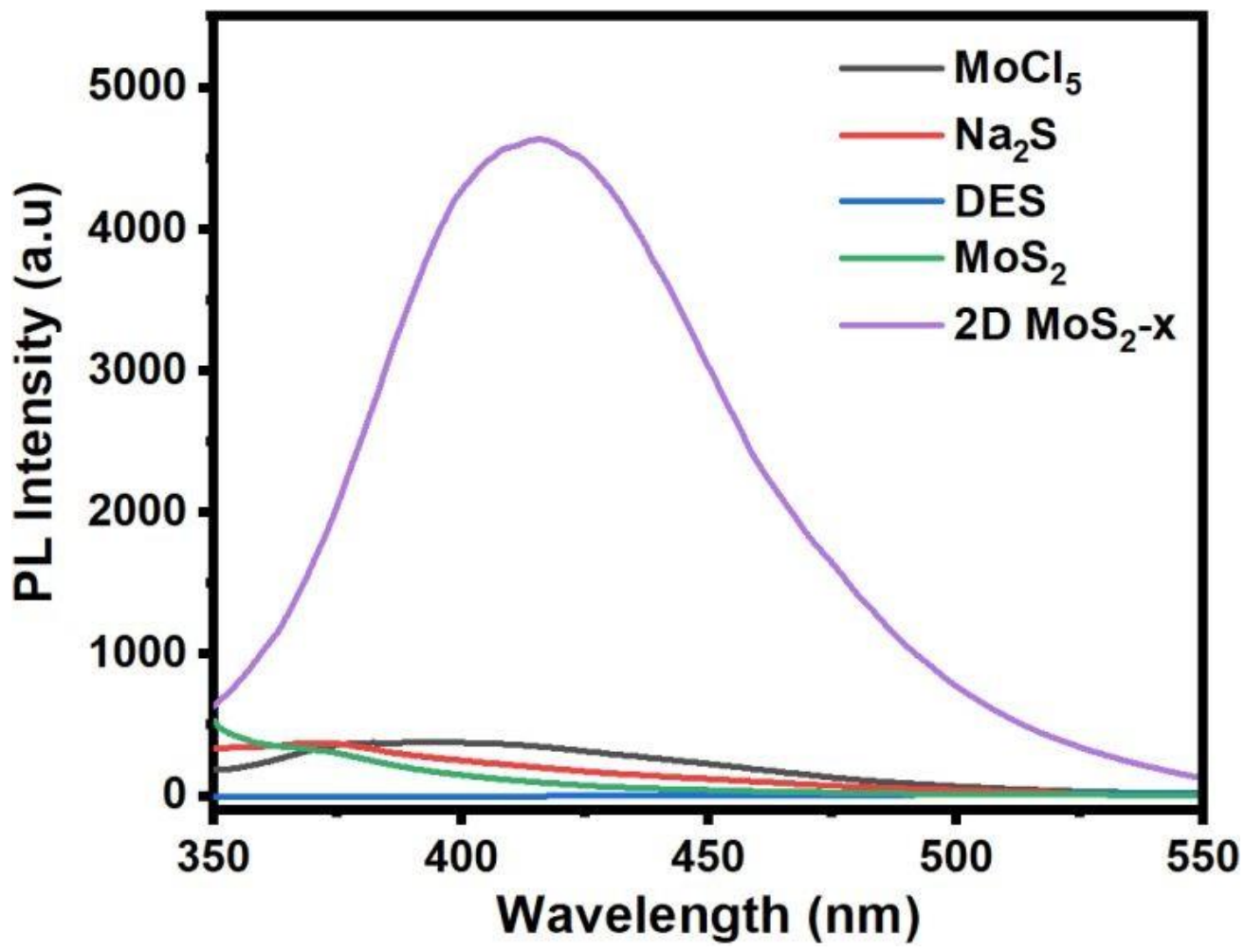

Figure S1. PL spectra of control experiments λ_{ex} = 330nm): (a) DES alone, no fluorescence; (b, c) $MoCl_5$ and Na_2S in water, no fluorescence; (d) Aqueous synthesis without DES, no fluorescence; (e) 2D MoS_{2-x} nanosheets, strong fluorescence at 420 nm.

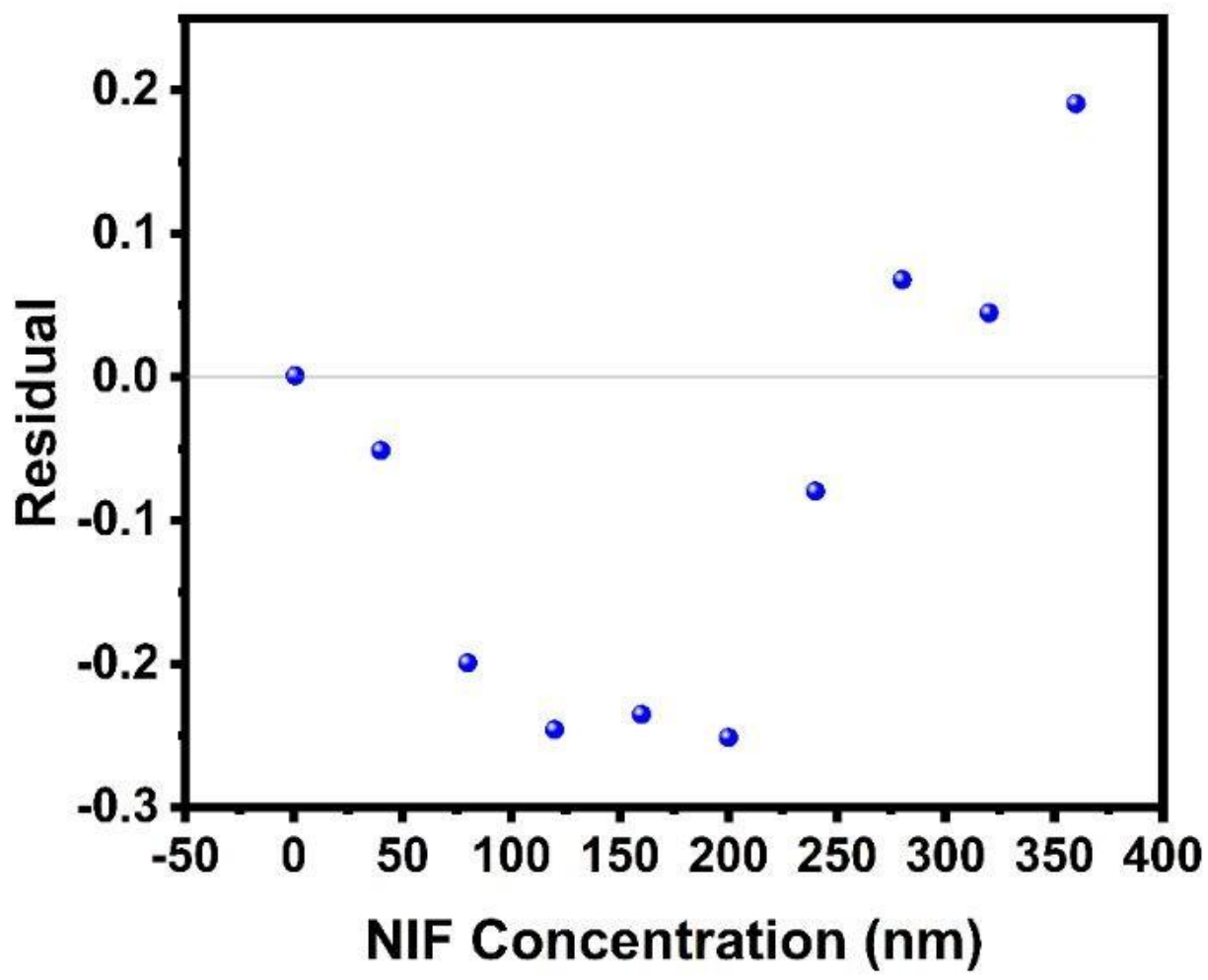

Figure S2. Residual plot for the calibration curve of NIF detection using 2D MoS_{2-x} nanosheets. Residuals are randomly scattered around the zero line with no obvious trend, confirming the suitability of the linear regression model.

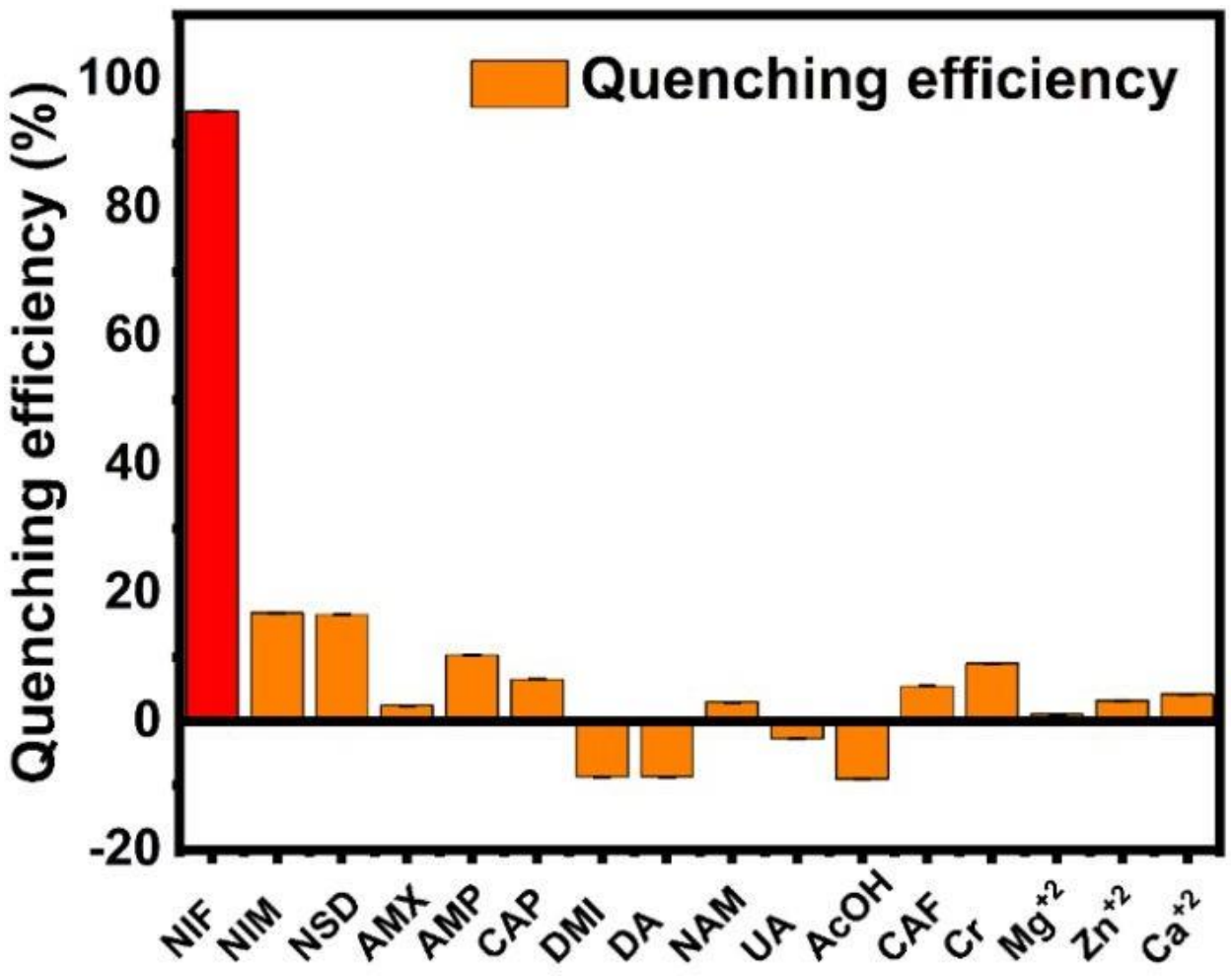

Figure S3. Comparative quenching efficiencies of NIF, its structural analogues, selected pharmaceutical drugs, and individual metal ions.

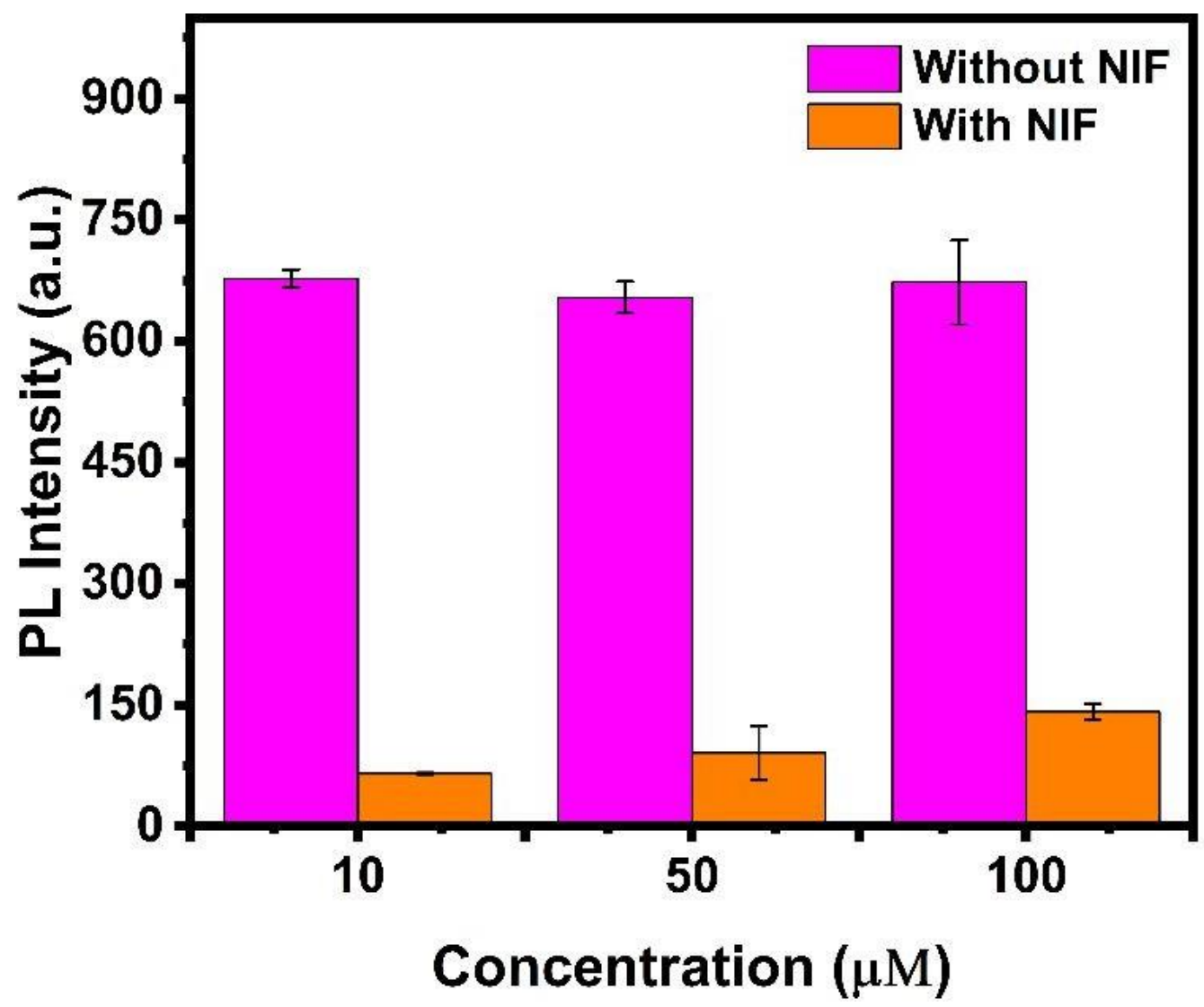

Figure S4. Fluorescence response of 2D MoS_{2-x} nanosensors in urine spiked with a mixture of interferents (10-100 μM) in the presence and absence of NIF (1 μM).

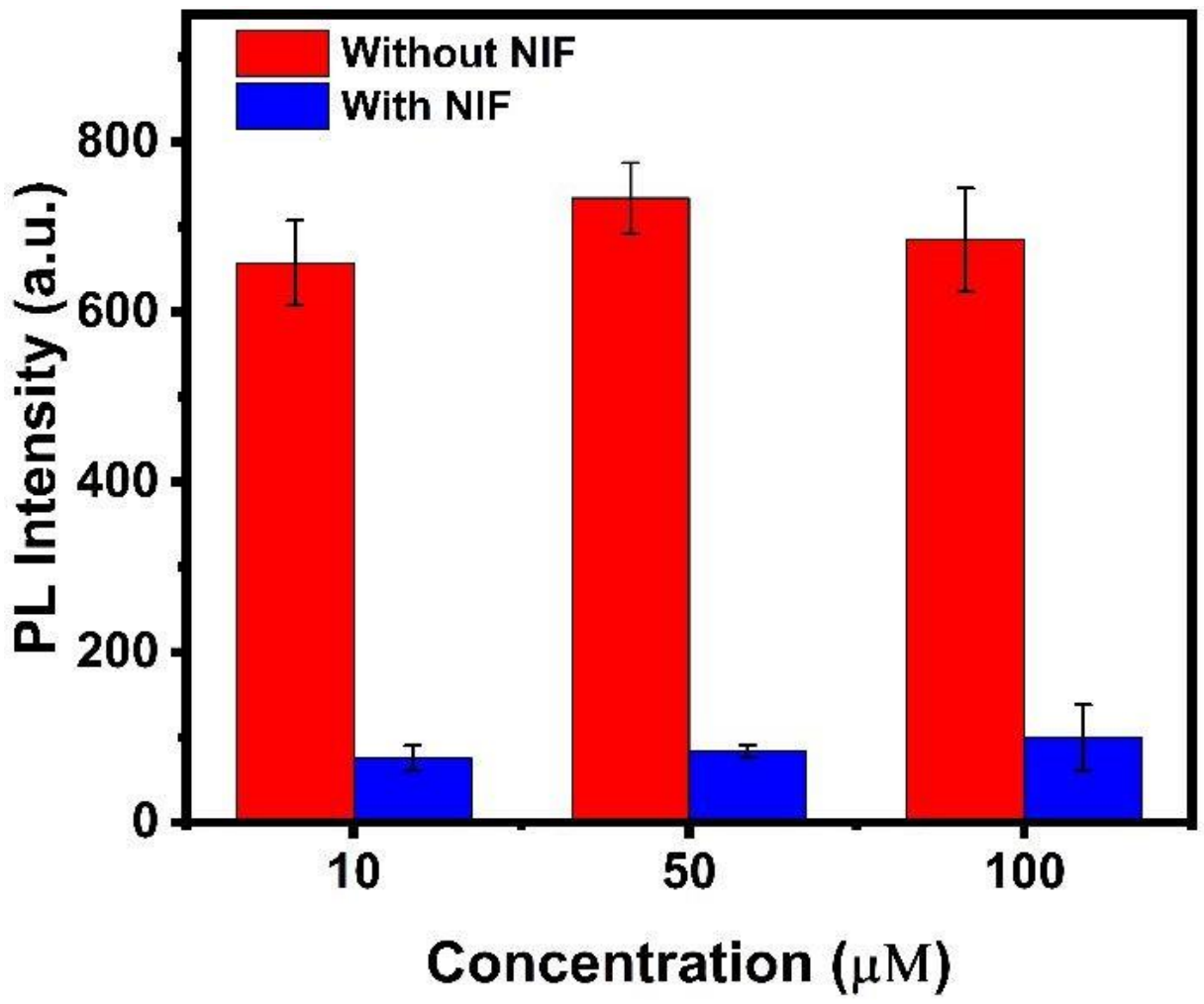

Figure S5. Fluorescence response of 2D MoS_{2-x} nanosensors in lotus pond water spiked with a mixture of interferents (10-100 μM) in the presence and absence of NIF (1 μM).

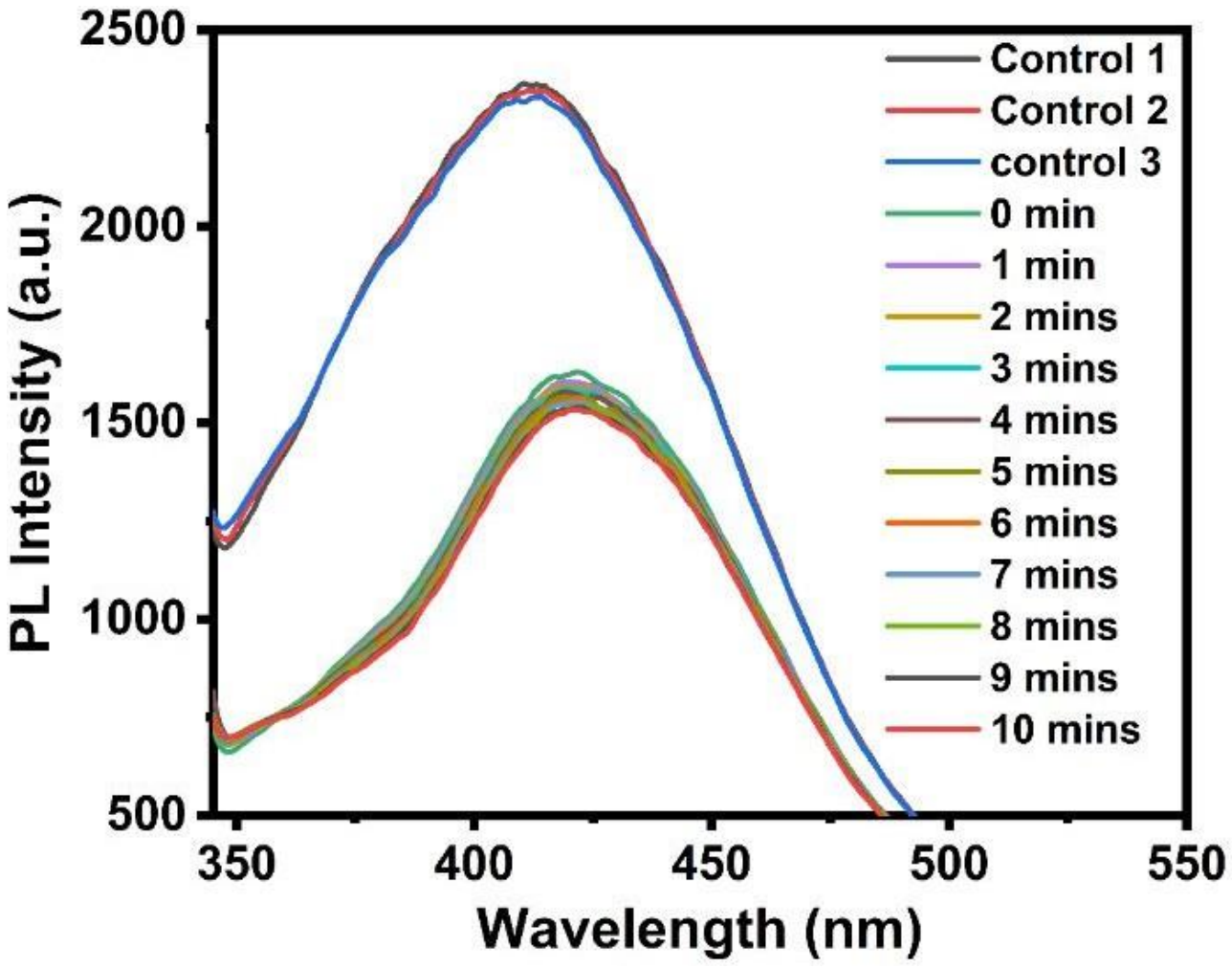

Figure S6. Response time analysis of the 2D MoS_{2-x} nanosensor upon addition of NIF (150 nM). Fluorescence intensity at 420 nm was monitored immediately after NIF addition. The quenching reached a stable plateau within <1 minute, indicating a rapid response suitable for real-time sensing applications.

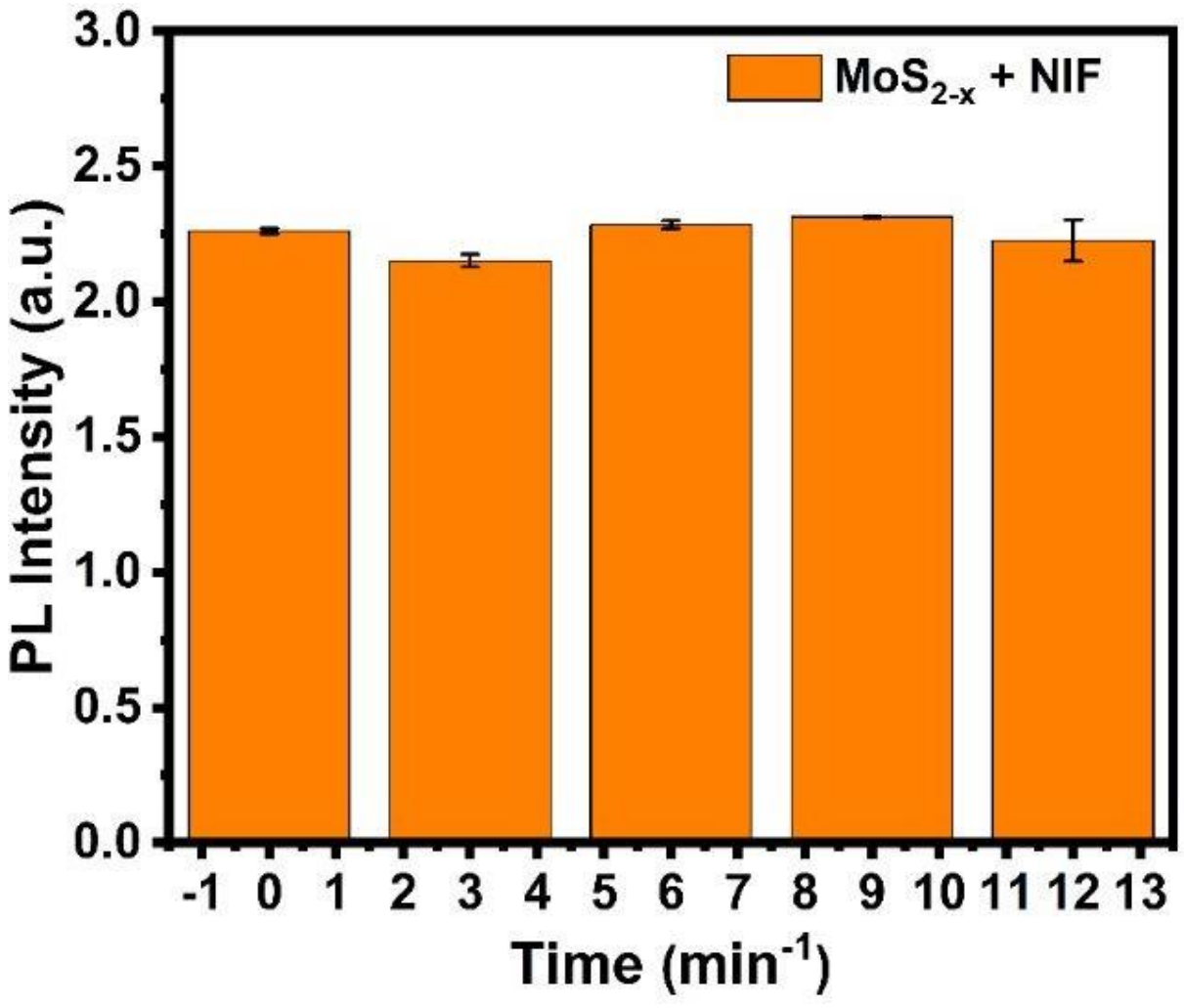

Figure S7. Intra-day fluorescence response of 2D MoS_{2-x} nanosheets upon NIF addition measured at different time intervals (hours) on the same day.

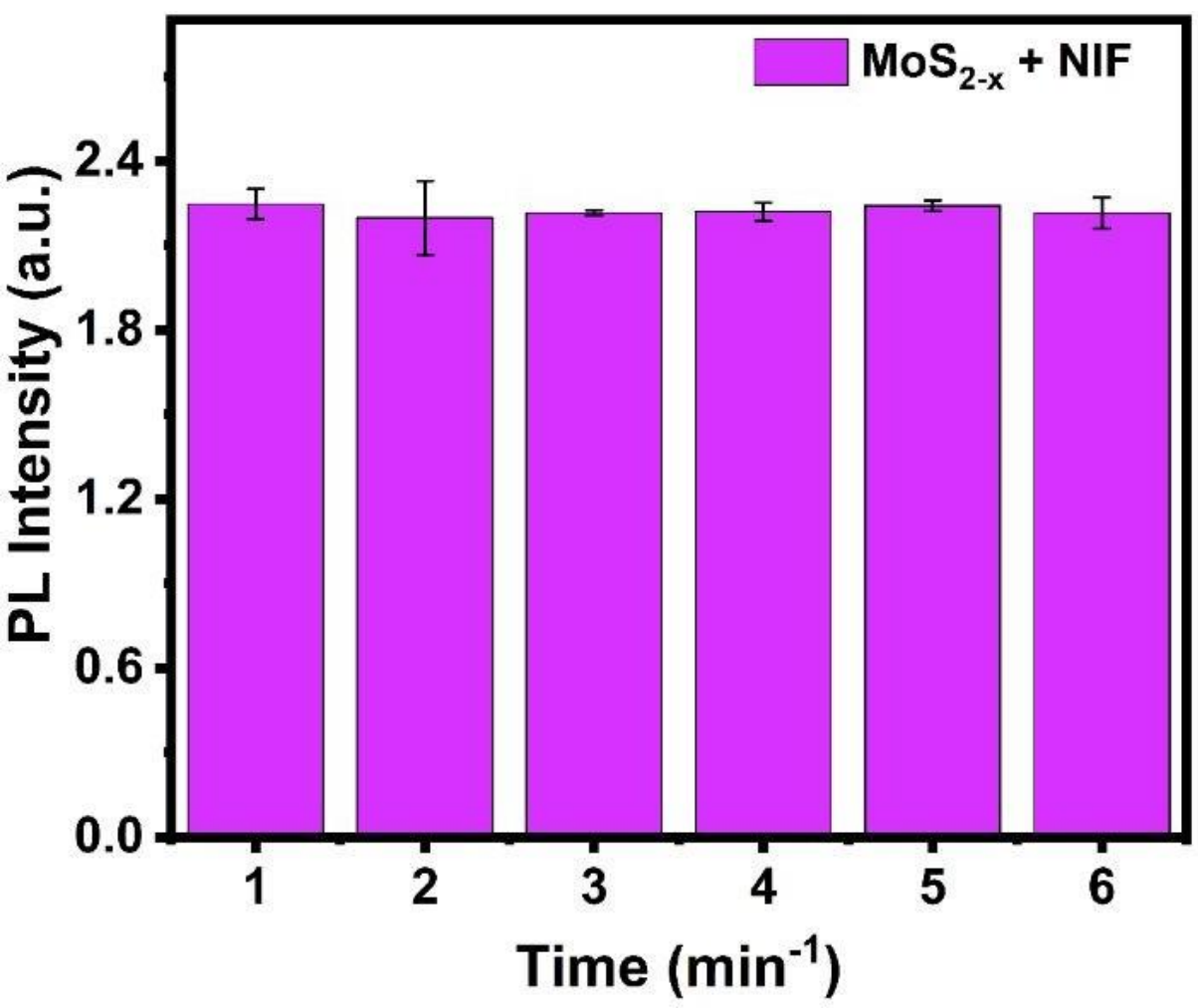

Figure S8. Inter-day fluorescence response of 2D MoS_{2-x} nanosheets upon NIF addition measured over different days.

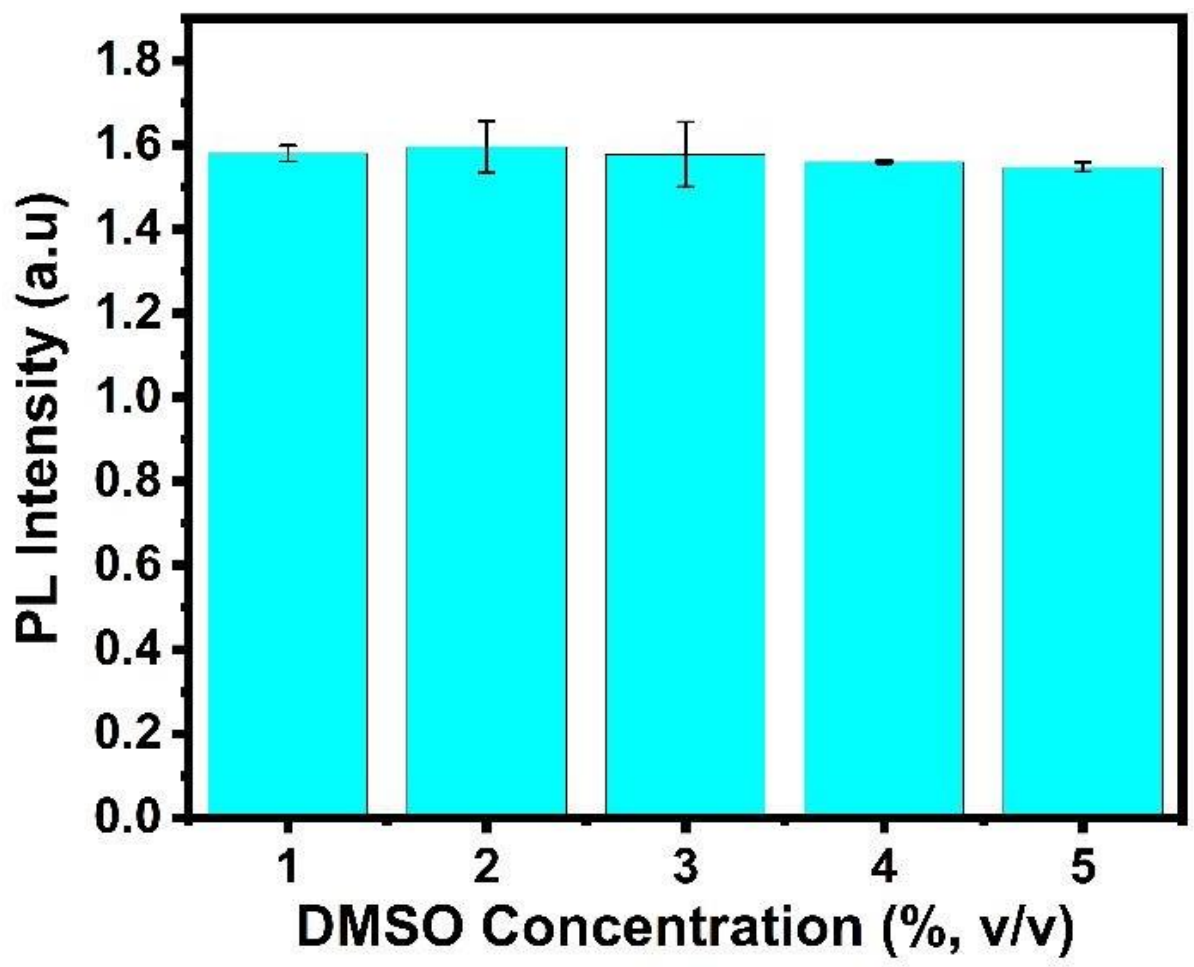

Figure S9. Effect of DMSO concentration (1—5%, v/v) on the fluorescence intensity of 2D MoS_{2-x} nanosheets, showing negligible interference

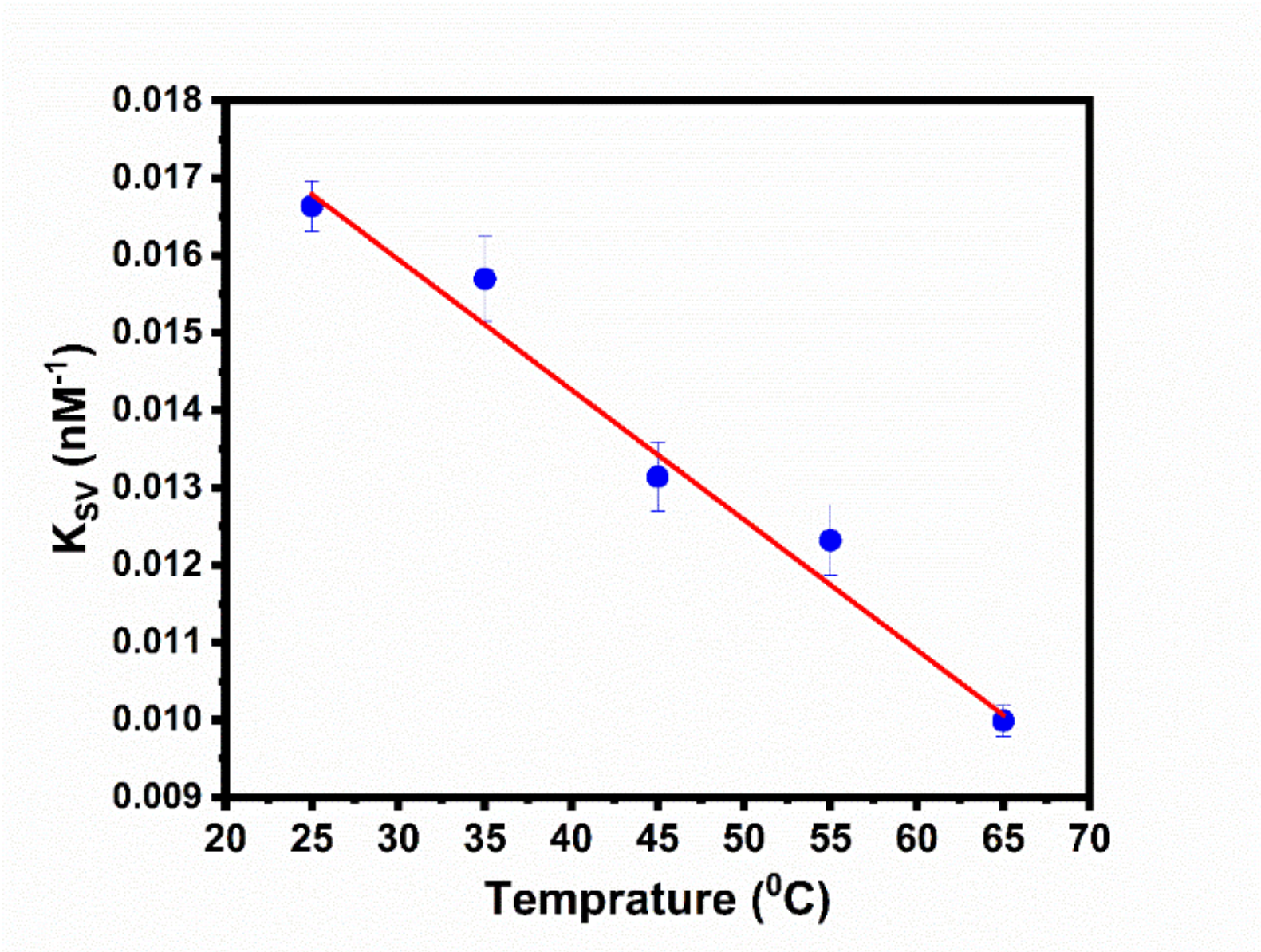

Figure S10. Temperature-dependent Stern-Volmer plot for the 2D MoS_{2-x} nanosheet-NIF system. The Stern-Volmer constant decreases from 0.01663 nM^{-1} at 25^{0}C to 0.0100 nM^{-1} at 65 ^{0}C, suggesting static quenching as the dominant mechanism. Error bars represent the standard deviation of triplicate measurements (n = 3).

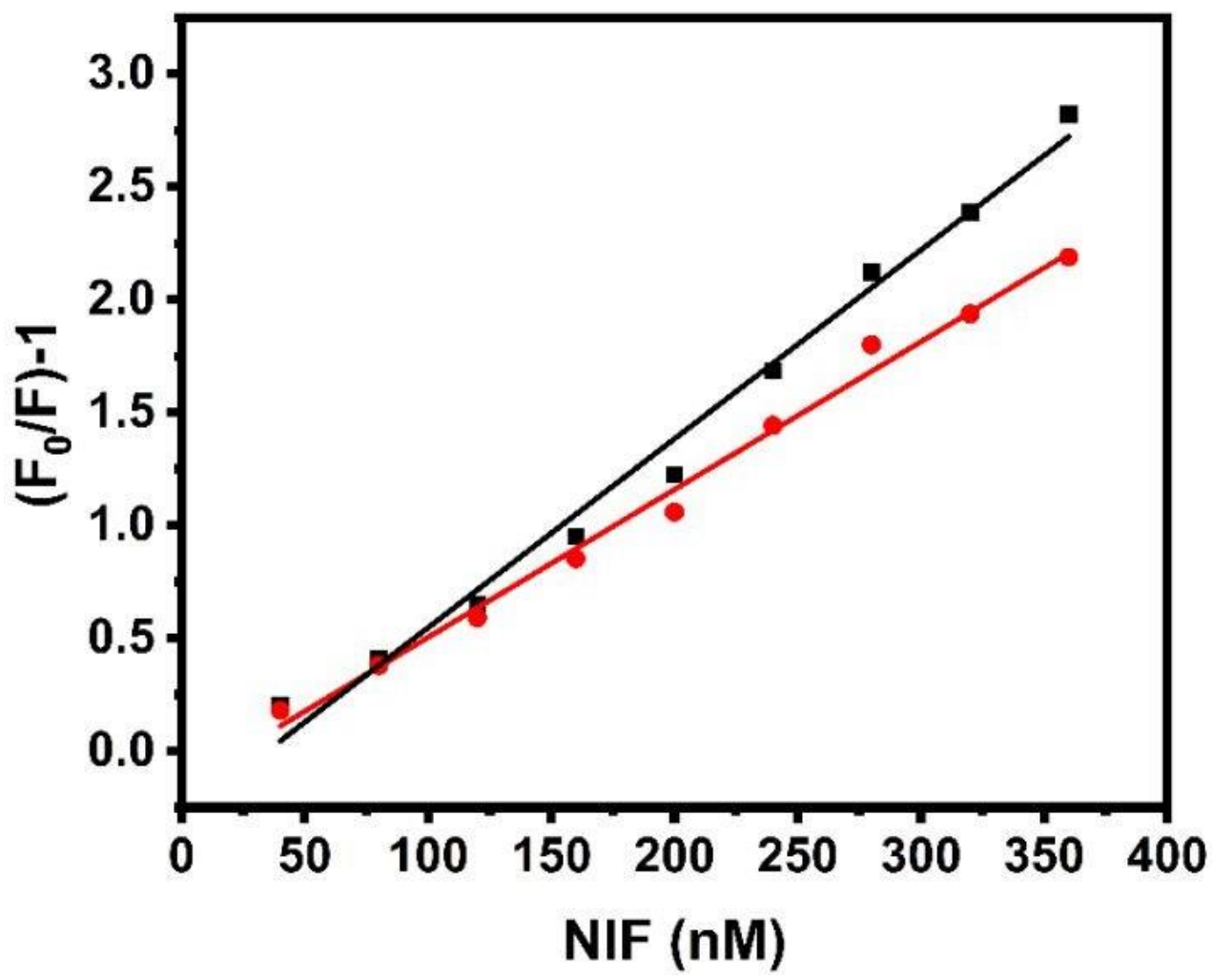

Figure S11. Stern-Volmer plots of MoS_{2-x} before (black) and after (red) quantitative IFE correction.

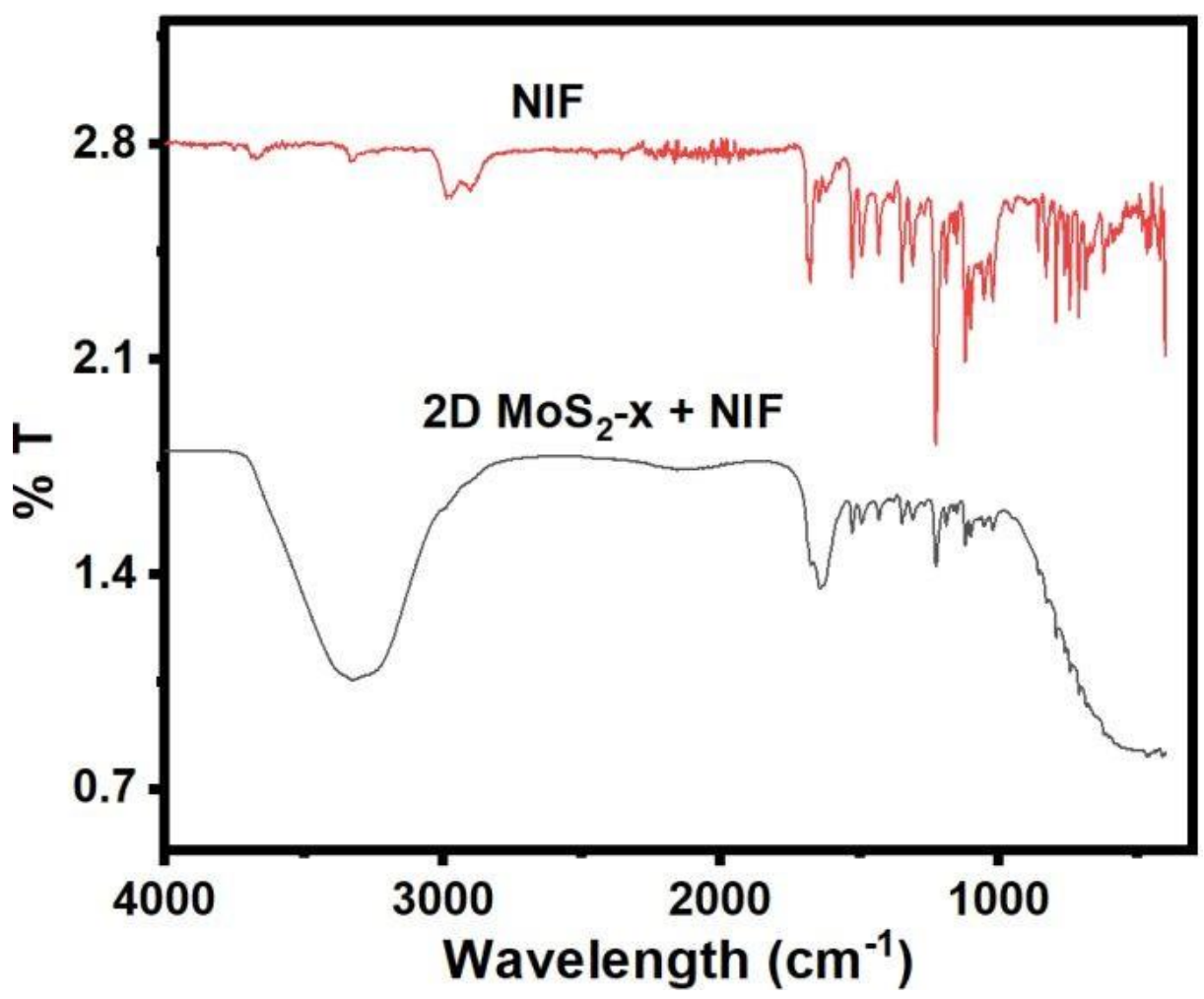

Figure S12. FTIR spectra of pristine NIF and 2D MoS_{2-x} nanosheets after interaction with NIF.

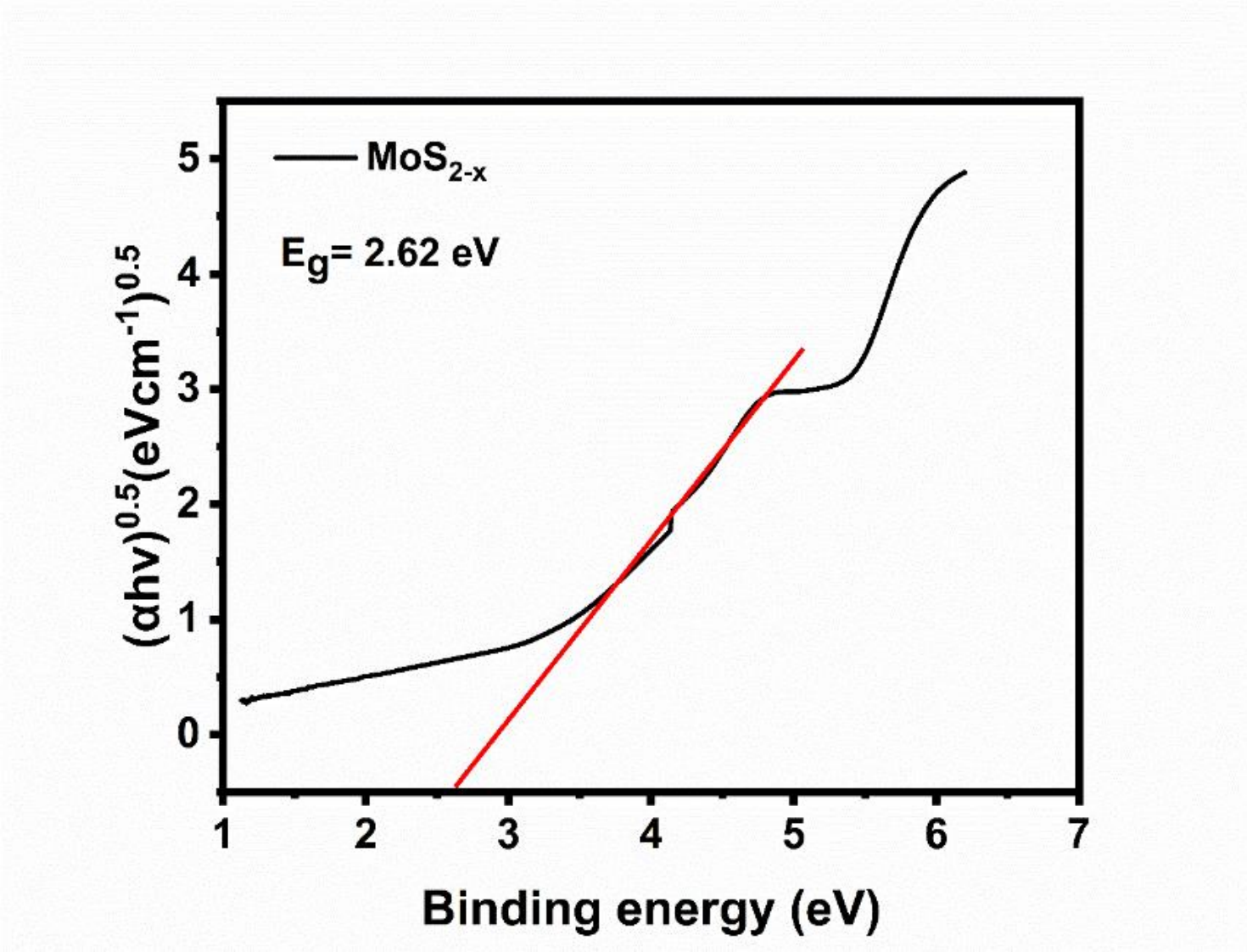

Figure S13. Tauc plot for indirect bandgap determination of pristine MoS_{2-x} nanosheets. The extrapolated linear fit gives an optical bandgap of 2.62 eV.

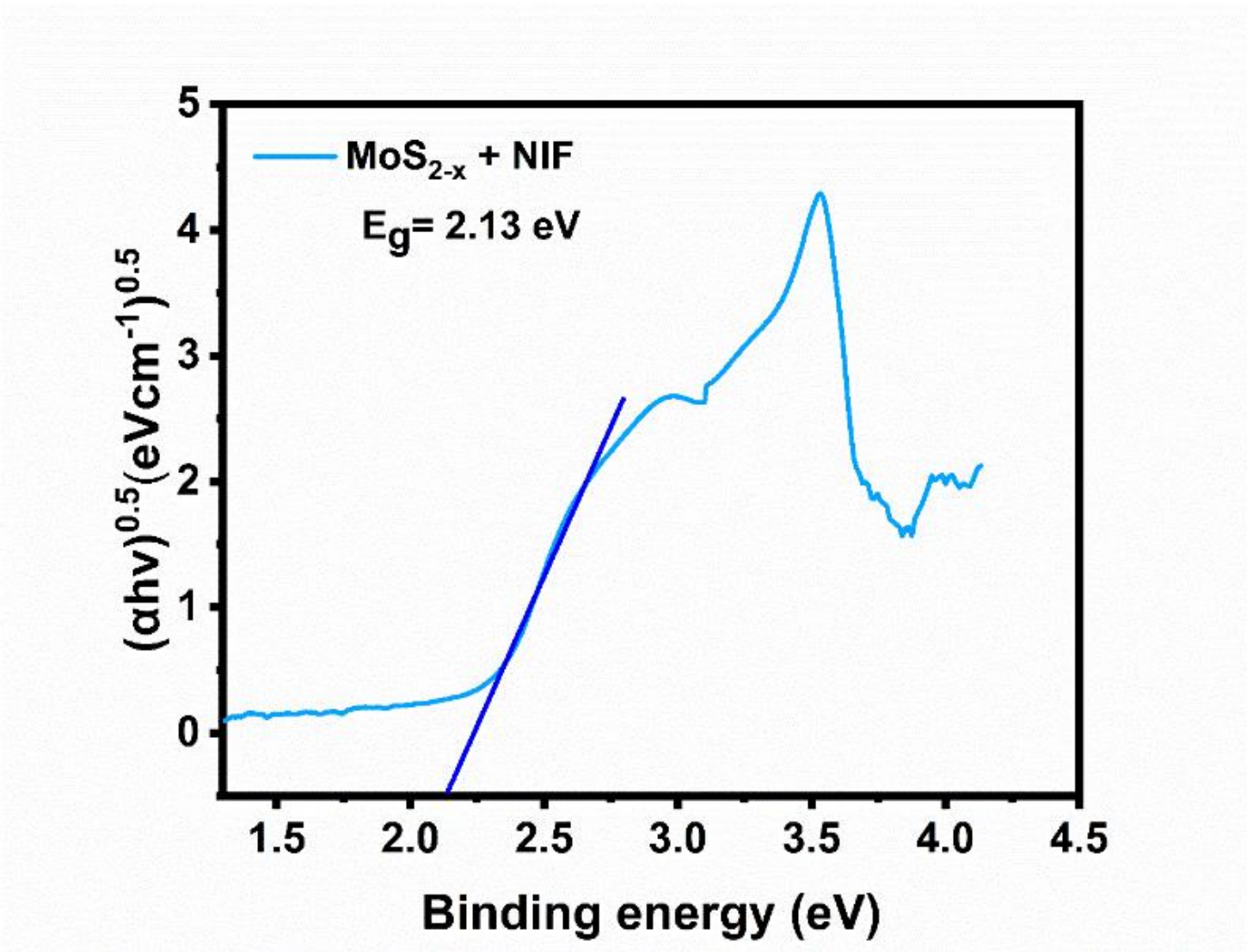

Figure S14. Tauc plot for indirect bandgap determination of MoS_{2-x} after interaction with NIF. The bandgap narrows to 2.13 eV, corresponding to a red shift of 0.49 eV, indicating electronic perturbation via charge transfer.

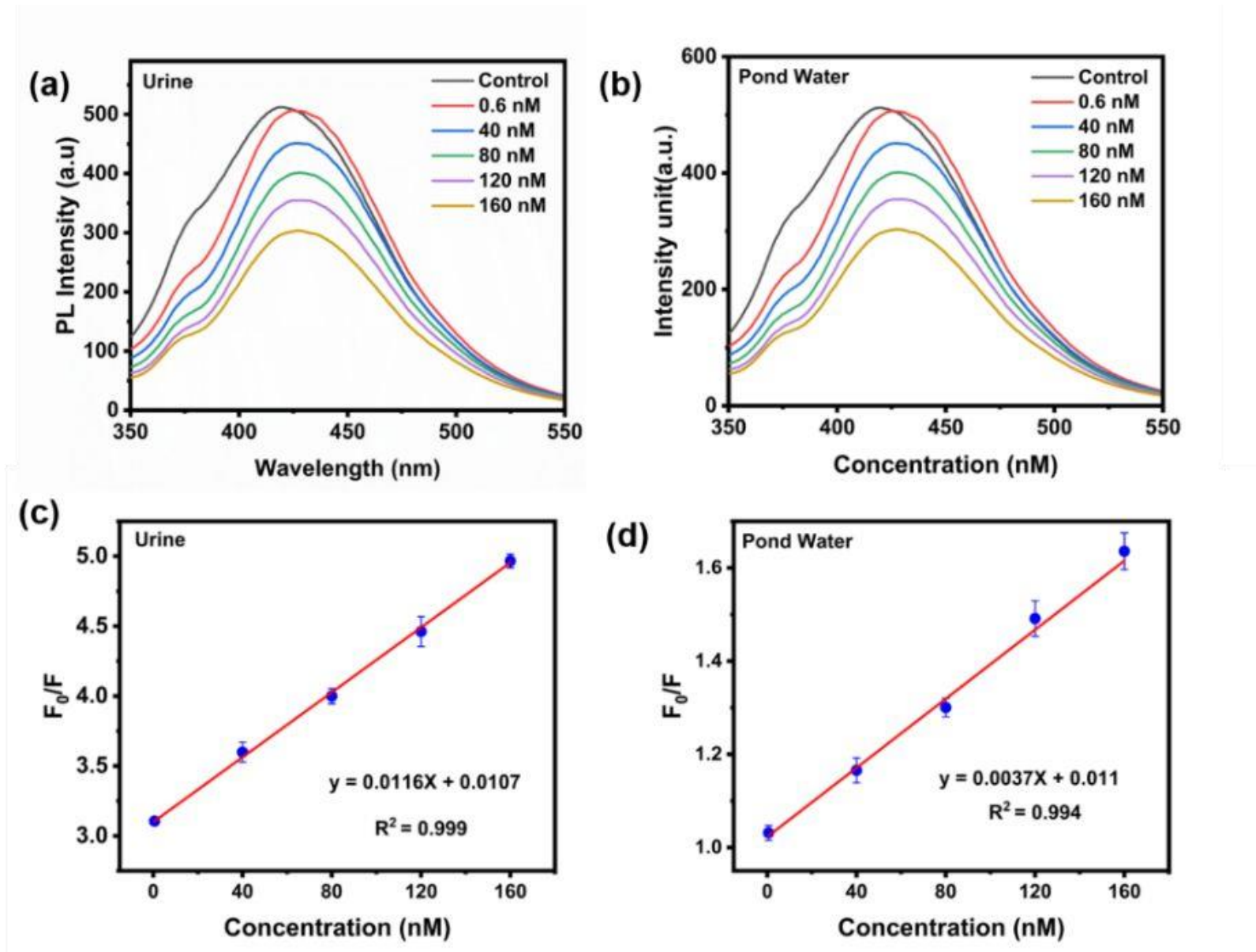

Figure S15. PL response of the 2D MoS_{2-x} for real sample analysis: (a) urine sample; (b) pond water; with corresponding linear calibration plots shown in (c) and (d), respectively.

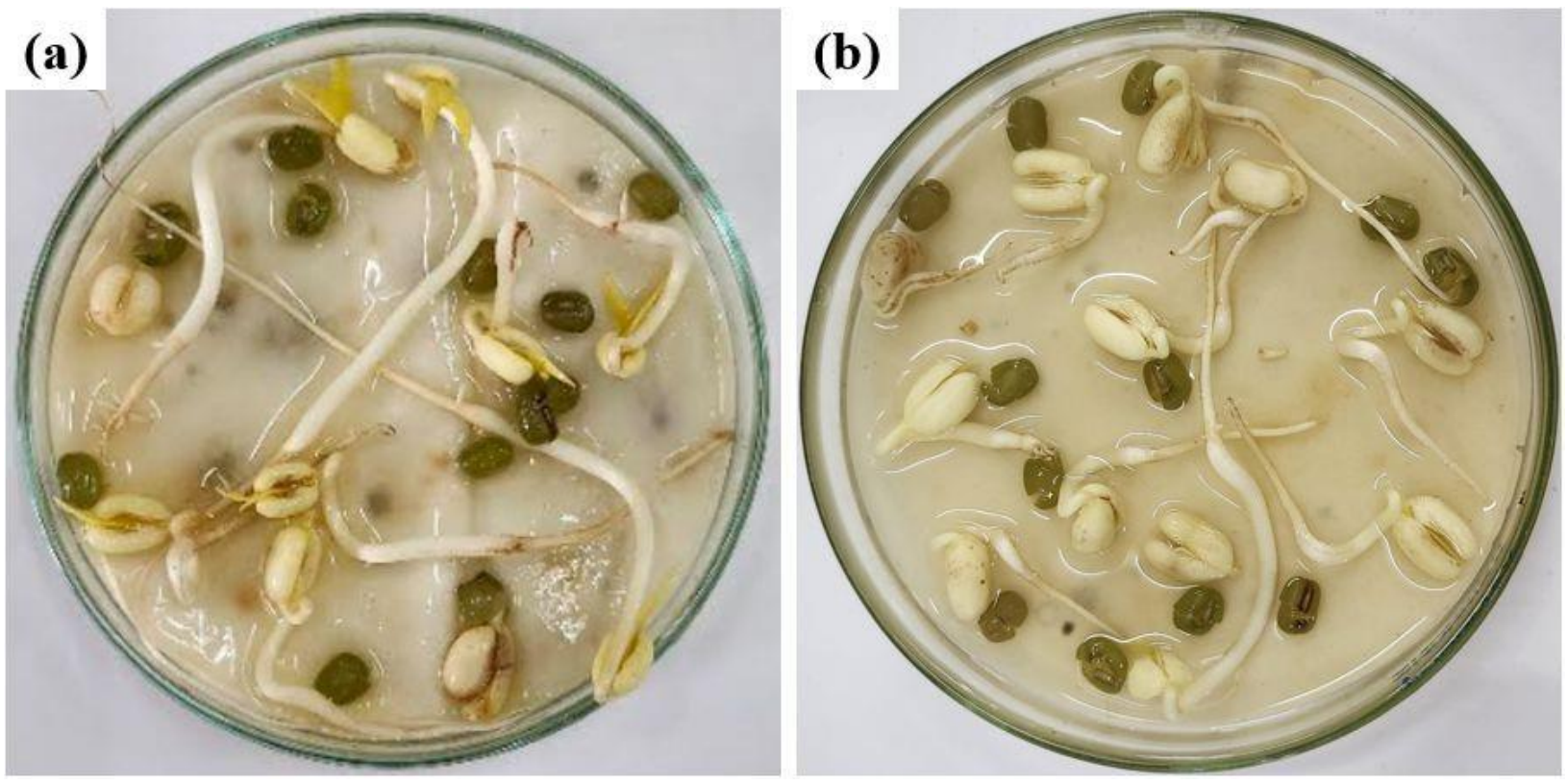

Figure S16. Mung bean seed germination test conducted in the dark: (a) Germination in tap water (control) after 5 days; (b) Germination in reaction residue (synthesis waste) after 5 days. Successful germination in the reaction residue confirms the non-phytotoxic nature of the DES-based synthesis waste.

Calculation section

S1. Statistical Validation and Limit of Detection (LOD) Calculation

The calibration curve for nifedipine (NIF) detection using 2D MoS_{2-x} nanosheets was validated through linear regression analysis. The statistical parameters, including the slope, intercept,

correlation coefficient, and 95% confidence intervals, are summarized in Table S1. A residual plot (Figure S2) was generated to confirm the appropriateness of the linear model; the residuals are randomly scattered around the zero line with no obvious trend, validating the linear regression for the concentration range of 0.6-360 nM. The detailed LOD calculation, based on ten replicate measurements of the blank solution, is provided in Table S2.

Table S1. Statistical parameters for the calibration curve of NIF detection using 2D MoS_{2-x} nanosheets.

Parameter	Value
Equation	$F^0/F = 1.03174 + 0.00722$ [NIF]
Linear range	0.6–360 nM
Slope (S)	0.00722 nM^{-1}
Standard error of slope	± 0.00021249 nM^{-1}
95% Confidence interval of slope	0.006747 to 0.007693 nM^{-1}
Intercept	1.03174
Standard error of intercept	± 0.00713
95% Confidence interval of intercept	1.01585 to 1.04763
Residual sum of squares	7.06143
Pearson's r	0.99656
R^2 (Coefficient of determination)	0.99313
Adjusted R^2	0.99227
Number of calibration points	10
Replicates per point	3
Blank replicates (n)	10
σ (Standard deviation of normalized blank)	0.00082
Limit of Detection (LOD = 3.3σ/S)	0.40 nM

Table S2. Blank replicates and LOD calculation for NIF detection using 2D MoS_{2-x} nanosheets.

Replicate	Raw Intensity (F^0)	F^0/F
1	513.7	1.0014
2	513.0	1.0000

3	512.9	0.9998
4	513.4	1.0009
5	513.3	1.0007
6	513.2	1.0004
7	512.8	0.9997
8	512.7	0.9994
9	512.6	0.9992
10	512.2	0.9985
**Mean (F^0)** = 512.98		
**Standard deviation of F^0/F (σ)** = 0.000851		
**Slope of calibration curve (S)** = 0.00722 nM^{-1}		

LOD Calculation

$$\text{LOD} = 3.3 \text{ x } \frac{\text{S. D}}{\text{Slope}}$$

LOD = 3.3 * 0.000851 / 0.00722 = 0.40 nM

Quenching efficiency

The fluorescence quenching efficiency (QE) of NIF toward 2D MoS_{2-x} nanosheets was calculated (Figure S3). using the following equation:

$$\text{QE}(\%) = \frac{I_0 - I}{I_0} \times 100$$

where I_0 and I are the fluorescence intensities of the nanosheets in the absence and presence of NIF, respectively. The observed fluorescence quenching is attributed to a combined mechanism of electron transfer and IFE arising from the spectral overlap between NIF absorption and the excitation/emission bands of the 2D MoS_{2-x} nanosheets.
